# Quantitative assessment of china’s policies on the online sale of prescription drugs

**DOI:** 10.1371/journal.pone.0358484

**Published:** 2026-09-23

**Authors:** Yuhang Zhang, Yuanyuan Zhang, Chenjian Zhao, Xingjuan Xu, Guoqing Wang, Xuefeng Xie

**Affiliations:** 1 School of Pharmacy, Anhui Medical University, Hefei Anhui, China; 2 Inflammation and Immune Mediated Diseases Laboratory of Anhui Province, Anhui Medical University, Hefei Anhui, China; 3 Anhui Provincial Drug Regulatory Scientific Research Center, Anhui Medical University, Hefei Anhui, China; 4 Key Laboratory of Public Health Social Governance, Philosophy and Social Sciences of Anhui Province, Anhui Medical University, Hefei Anhui, China; McGill University Faculty of Arts, CANADA

## Abstract

Against the dual backdrop of the rapid advancement of big data-driven healthcare and the COVID-19 pandemic, China has gradually relaxed restrictions on the online sale of prescription drugs. However, the regulatory framework governing this sector remains in its early stage of development, and existing policy gaps pose substantial risks to public medication safety, highlighting the urgent need for further policy refinement. This study quantitatively analyzed China’s policies on the online sale of prescription drugs, thus providing valuable insights for the development and improvement of future policies. Using ROST CM6.0, text mining was conducted on 18 policies governing the online sale of prescription drugs issued between 1999 and 2023. A Policy Model Consistency (PMC) model was established based on 10 primary variables and 35 secondary variables. The analysis yielded an average PMC index of 6.28, resulting in an overall policy rating of “good.” Among the 18 policies, 6 were classified as excellent and 12 as good, with no policies rated as perfect or unqualified. Amid the continuous exploration of policy and reforms, China’s policy system for the online sale of prescription drugs has been gradually improved. Current policies place strong emphasis on the efficiency of smart approaches and the steady development of an intelligent regulatory system. However, considerable opportunities for optimization remains in key areas, including the whole-process regulatory system for the online sale of prescription drugs, the collaborative participation of medical institutions, and the standardized governance of third-party platforms. Future policies should focus on the regulations governing the online sale of prescription drugs by clarifying the boundaries, rights and responsibilities of each subject while refining and standardizing their code of conduct. Multidepartment collaboration should also be strengthened by encouraging industry associations in the development of an intelligent regulatory team, thereby promoting the establishment of a three-dimensional digital regulatory pattern.

## 1. Introduction

The online sale of medicinal products first emerged in the United States in the late 1990s [1]. Driven by rapid advancements in Internet technology and the widespread impact of the COVID-19 pandemic, the number of online pharmacies has been experiencing a stable year-on-year growth, making the procurement of prescription medications via digital platforms an increasingly common and potentially long-term consumer behavior [2–4]. The low cost, high accessibility, and robust privacy protection offered by online pharmacies have substantially enhanced consumers’ propensity to purchase pharmaceuticals online [2,5]. However, the online procurement of prescription medications also presents considerable risks to public health. For example, certain commercial entities reap exorbitant profits by marketing counterfeit or substandard pharmaceutical products. Moreover, the absence of standardized instructions regarding the appropriate administration of medications increase the risk of inappropriate self-medication and misuse of prescription drugs among consumers [6–8]. Orizio Grazia et al. hold the view that formulating and implementing policies to establish a comprehensive regulatory framework can effectively mitigate the risks associated with the online purchase of prescription medications [1]. Aiming to protect the legitimate rights and interests of consumers, the United States has successively enacted and promulgated a series of regulatory measures, including the Federal Food, Drug, and Cosmetic Act, the Internet Drug Sales Action Plan, and the Ryan Haight Online Pharmacy Consumer Protection Act. These regulations have strengthened and refined the policy regulatory system governing the online sale of prescription drugs [9,10].

The regulatory policies of China regarding the online sale of prescription drugs have undergone substantial evolution over the past two decades. Initially characterized by stringent bans, the regulatory approach shifted toward a phase of sustained pilot initiatives and has since progressed to a more cautious and detailed regulation [11]. These policies, encompassing guidelines and drafts released for public consultation, have played a pivotal role in ensuring the stability of the online sale market of prescription drugs and promoting its advancement. As a crucial component of public policy analysis, policy evaluation provides an important basis for subsequent policy refinement and the resolution of pertinent issues through its scientific and systematic assessment outcomes [12,13]. However, to date, no study has employed a comprehensive and scientifically rigorous methodology to analyze China’s policies governing the online sale of prescription medications. This paper utilizes text mining techniques and, using the Policy Modeling Consistency (PMC) Index model, performs a quantitative assessment of relevant policies governing the online sale of prescription drugs in China. The aim is to provide useful references for the government in devising ideal and effective policies pertaining for the future advancement of this sector.

## 2. Data sources and research methods

### 2.1. Data sources

The official websites of government departments, including the National Medical Products Administration, the National Administration for Market Regulation, and the National Development and Reform Commission, were used to retrieve policy documents. The search was conducted using keywords such as “prescription drugs,” “Internet,” and “online drug sales.” Relevant documents whose policy content was consistent with the research theme of this study were screened. A total of 18 policy documents were selected for analysis, as shown in Table 1.

**Table 1:** Policy texts for the online sale of prescription drugs.

Code	Release time	Policy name
P1	December 1999	Interim Regulations on the Circulation Governance of Prescription Medications and Over-the-Counter Medications
P2	September 2005	Interim Provisions on the Examination and Approval of Internet-based Pharmaceutical Trading Services
P3	January 2007	Measures for the Supervision and Administration of Circulation of Pharmaceuticals
P4	May 2011	Notice on Further Strengthening the Crackdown on the Illegal Sale of Drugs and the Dissemination of False Pharmaceutical Information via the Internet
P5	October 2013	Notice of the China Food and Drug Administration on Strengthening the Administration of Internet Drug Sales
P6	May 2014	Regulations on the Supervision and Administration of Internet Food and Drug Operations (Draft for Comment)
P7	July 2015	Guiding Opinions on Actively Promoting the “Internet Plus” Initiative
P8	April 2017	Notice of the General Office of the China Food and Drug Administration on the Work concerning the Implementation of the Decision of the State Council on Canceling the Third Group of Administrative Licensing Items Designated by the Central Government for Implementation by Local Governments
P9	November 2017	Measures for the Supervision and Administration of Online Pharmaceutical Operations (Draft for Comments)
P10	August 2019	Drug Administration Law of the People’s Republic of China (2019 Revision)
P11	November 2020	Measures for the Supervision and Administration of Online Sale of Medicinal Products (Draft for Comments)
P12	April 2021	Opinions of the National Development and Reform Commission and the Ministry of Commerce on Several Special Measures for Supporting the Construction of the Hainan Free Trade Port and Relaxing Market Access
P13	April 2021	Opinions of the General Office of the State Council on Supporting Efforts to Ensure Stability on Six Fronts and Maintain Security in Six Areas and Further Promoting the Reform of “Simplification of Administrative Procedures, Decentralization of Powers, Combination of Decentralization with Appropriate Control, and Optimization of Services”
P14	January 2022	Opinions of the National Development and Reform Commission and the Ministry of Commerce on Several Special Measures for Relaxing Market Access in Shenzhen’s Building of a Pioneering Demonstration Zone for Socialism with Chinese Characteristics
P15	August 2022	Measures for the Supervision and Administration of Online Sale of Medicinal Products
P16	November 2022	Notice by the Department of Comprehensive Affairs, Planning, and Finance Affairs of the National Medical Products Administration of Effectively Implementing the Measures for the Supervision and Administration of the Online Sale of Medicinal Products
P17	February 2023	Measures for the Administration of Internet Advertising
P18	June 2023	Notice on Regulating the Information Display of Prescription Drugs in Online Sales

### 2.2. Analysis of policy text mining

Text mining is a knowledge-intensive process that facilitates the extraction of valuable information from unstructured text data while enabling the assessment of the quality and pertinence of the derived results [14–16]. In this study, ROST CM6.0 software was used to conduct in-depth text mining analysis of the original Chinese versions of policies governing the online sale of prescription drugs. The results serve as a fundamental basis for parameter construction within the PMC index model, thereby supporting a highly refined and systematic evaluation framework in this domain.

### 2.3. Research methods

Scientifically sound and well-calibrated policy evaluation is crucial for national development [12]. The Omnia Mobilis Hypothesis postulates that all entities exist in a state of perpetual motion and are intricately interconnected. In this context, policy modeling emerges as a powerful analytical approach with broad applicability across an extensive spectrum of disciplines. Therefore, effective policy modeling should incorporate a comprehensive set of variables than focusing on merely beneficial variables.

In 2011, Estrada introduced the PMC Index model [17]. This model enables the scientific assessment of the consistency level within policy modeling frameworks and facilitates the identification of the strengths and weaknesses of individual policies. The policy modeling process can be systematically delineated into the following sequential steps:

① Variable selection and parameter identification;
② Construction of a multi-input and multi-output table;
③ Computation of the PMC Index value;
④ Generation of a surface chart based on the calculated PMC Index value.

### 2.4. Latent dirichlet allocation

Latent Dirichlet Allocation (LDA) is an unsupervised generative probabilistic model that is widely applied to topic modeling of textual data [18]. This model identifies the topic distributions contained in texts by determining the corresponding word distributions for each individual topic. In this study, an LDA model was implemented in Python to analyze 18 policies concerning the online sale of prescription drugs, aiming to identify the underlying thematic structure and examine the evolutionary trends of these policies.

### 2.5. Entropy weight method

The entropy weight method is an objective weighting technique based on the principle of information entropy. Its core idea is to determine the weight of each indicator according to its degree of dispersion, thereby reflecting the amount of information contained in the data [19]. In this study, the entropy weight method was adopted to assign objective weights to the first-level variables, avoiding potential biases caused by subjective assignment and improving the objectivity and scientific rigor of the evaluation results. The main steps are as follows:

① Construct the evaluation matrix and standardize the data;
② Calculate the information entropy;
③ Calculate the difference coefficient based on the information entropy;
④ Compute the weight of each indicator and conduct corresponding analysis.

## 3. Construct the PMC index model

### 3.1 Variable selection and parameter identification

**Fig 1:**
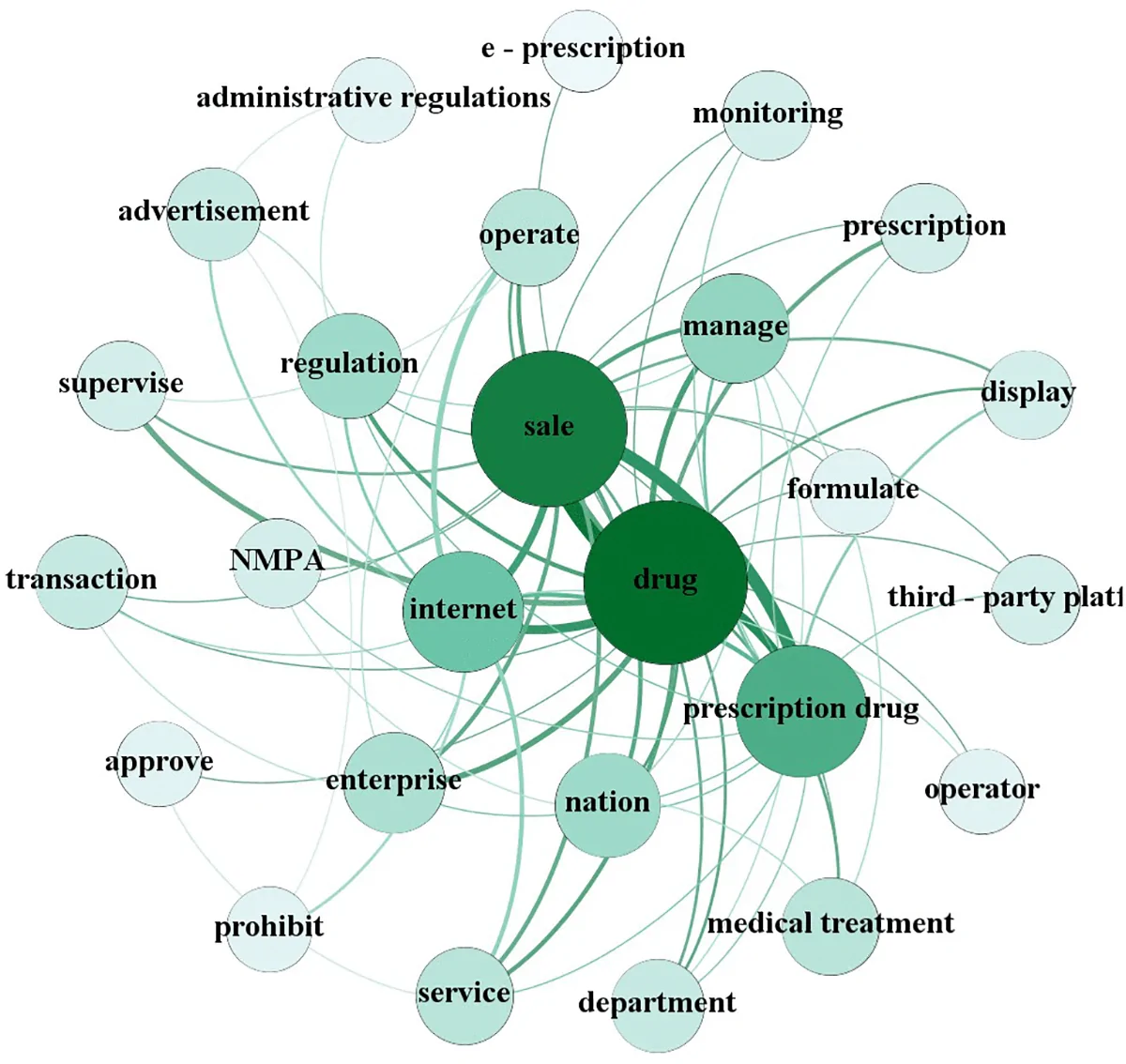
Keyword Social Network Graph of Policies Governing the Online Sale of Prescription Drugs.

Leveraging ROST CM6.0 software, text mining was performed on 18 policies governing the online sale of prescription drugs. Following pre-processing procedures, including lexical segmentation, word frequency analysis, and the extraction of high-frequency terms, a co-occurrence network graph of high-frequency words was constructed, as depicted in Fig 1 [20]. Although network analysis is effective for visualizing relational structures, the PMC Index Model offers a more robust and nuanced framework for the quantitative evaluation of policy effectiveness. Furthermore, the results of network analysis provide objective empirical support for the construction of the PMC index system. Therefore, this study adopts the PMC Index Model to conduct a systematic quantitative assessment of policies governing the online sale of prescription drugs.

Incorporating the co-occurrence network diagram of high-frequency terms, findings from pertinent academic literature, and expert insights, a comprehensive policy quantitative assessment index system was meticulously constructed [13,21–23]. Among these indicators, X2, X3, X4, and X10 respectively represent policy constraint intensity, issuing authority tier, legal validity level, and policy disclosure requirements, which serve as core dimensions for assessing the legality and authority of regulatory policies governing the online sales of prescription drugs. Aiming to ensure the integrity and rigor of the evaluation system and perform comprehensive quantitative assessments of the included policies, this study established a multidimensional quantitative policy evaluation framework comprising 10 primary variables and 35 secondary variables, as detailed in Table 2.

**Table 2:** Quantitative Evaluation Index System of 18 Policies.

Primary variable	Secondary variable numbering	Secondary variable name	References
X1 Policy nature	X1:1	provision	[22]
	X1:2	notification	
	X1:3	advice	
X2 Policy stance	X2:1	prohibit	expert advice
	X2:2	authorize	
X3 Issuing institution	X3:1	National medical products administration	[13] and text
	X3:2	State administration for market regulation	
	X3:3	the State Council	
	X3:4	The national people’s congress of the people’s republic of china	
	X3:5	National development and reform commission	
X4 Validity level	X4:1	law	[23]
	X4:2	administrative regulations	
	X4:3	Departmental regulations	
	X4:4	Departmental working documents	
X5 Policy object	X5:1	government institution	[22] and text
	X5:2	Internet drug operators	
	X5:3	medical institution	
	X5:4	third – party platform	
	X5:5	others	
X6 Policy function	X6:1	supervision and restraint	[13] and text
	X6:2	standardize and guide	
	X6:3	guidance	
X7 Policy importance	X7:1	The policy content accounts for more than 0.2% of the total length.	expert advice and text
	X7:2	The policy content accounts for more than 5% of the total length.	
	X7:3	The policy content accounts for more than 10% of the total length.	
X8 Policy perspective	X8:1	macro	[21]
	X8:2	micro	
X9 Policy content	X9:1	access supervision	[13] and text
	X9:2	quality supervision	
	X9:3	punitive measures	
	X9:4	transaction supervision	
	X9:5	Advertising supervision	
	X9:6	distribution supervision	
	X9:7	information supervision	
X10 Policy disclosure	X10:1		

### 3.2 Calculation of PMC index values

The PMC index values for the 18 policies were calculated based on the quantitative evaluation index system and corresponding evaluation criteria for policies governing the online sale of prescription drugs. The calculation of the PMC index was performed using the following four-step process [24]:

① Incorporate the first- and second-order variables into a multi-input and multi-output table.

② Set the parameters to either 0 or 1. A value of 1 was assigned if the policy text satisfied the requirements of the corresponding second-order variable; otherwise, a value of 0 was assigned.

③ Compute the values of the first-order variables using Formula (1). These values should fall within the range of [0, 1], where T(X_ij_) denotes the number of second-order variables corresponding to a first-order variable, with i and j representing the first- and second-order variables, respectively.

④ Determine the PMC index for each policy using Formula (2).

Based on the two core cluster centers (5.81, 6.75), which were derived from cluster analysis of PMC index results using SPSS 27.0, and policy evaluation criteria proposed in prior literature [23,25], this study developed a grading standard for the PMC index: PMC index values from 8.50 to 10.00 correspond to “Perfect”, 6.50–8.49 to “Excellent”, 5.00–6.49 to “Good”, and 0.00–4.99 to “Non-compliant”. To test the robustness of this grading standard, two alternative schemes with moderately adjusted cut-points around the baseline critical values were constructed for sensitivity analysis. Scheme A: Perfect [8.30, 10.00], Excellent [6.30, 8.29], Good [4.80, 6.29], Non-compliant [0.00, 4.79]; Scheme B: Perfect [8.70, 10.00], Excellent [6.70, 8.69], Good [5.20, 6.69], Non-compliant [0.00, 5.19]. The results indicate that three policies experience grade shifts under Scheme A, while only one policy undergoes a grade adjustment under Scheme B. No samples fall into the Perfect or Non-compliant intervals. The low proportion of sample reclassification across alternative schemes demonstrates that the defined grading bands exhibit satisfactory robustness. Therefore, the original threshold set is adopted as the grading criterion for policy evaluation in this study.


$$\begin{array}{c} {\text{X}}_{\text{i}}=\left[\sum_{\text{i}=\text{1}}^{\text{n}}\frac{{\text{X}}_{\text{ij}}}{\text{T}\left({\text{X}}_{\text{ij}}\right)}\right] \end{array}$$ (1)

$$\begin{array}{cc} \text{PMC}= & \sum_{\text{i}=\text{1}}^{\text{10}}\left({\text{X}}_{\text{i}}\left[\sum_{\text{i}=\text{1}}^{\text{n}}\frac{{\text{X}}_{\text{ij}}}{\text{T}\left({\text{X}}_{\text{ij}}\right)}\right]\right)=[{\text{X}}_{\text{1}}\left(\sum_{\text{i}=\text{1}}^{\text{3}}\frac{{\text{X}}_{\text{1i}}}{\text{3}}\right)+{\text{X}}_{\text{2}}\left(\sum_{\text{j}=\text{1}}^{\text{2}}\frac{{\text{X}}_{\text{2j}}}{\text{2}}\right)+{\text{X}}_{\text{3}}\left(\sum_{\text{k}=\text{1}}^{\text{5}}\frac{{\text{X}}_{\text{3k}}}{\text{5}}\right)+{\text{X}}_{\text{4}}\left(\sum_{\text{l}=\text{1}}^{\text{4}}\frac{{\text{X}}_{\text{4l}}}{\text{4}}\right)+{\text{X}}_{\text{5}}\left(\sum_{\text{m}=\text{1}}^{\text{5}}\frac{{\text{X}}_{\text{5m}}}{\text{5}}\right)\\ & +{\text{X}}_{\text{6}}\left(\sum_{\text{n}=\text{1}}^{\text{3}}\frac{{\text{X}}_{\text{6n}}}{\text{3}}\right)+{\text{X}}_{\text{7}}\left(\sum_{\text{o}=\text{1}}^{\text{3}}\frac{{\text{X}}_{\text{7o}}}{\text{3}}\right)+{\text{X}}_{\text{8}}\left(\sum_{\text{p}=\text{1}}^{\text{2}}\frac{{\text{X}}_{\text{8p}}}{\text{2}}\right)+{\text{X}}_{\text{9}}\left(\sum_{\text{q}=\text{1}}^{\text{7}}\frac{{\text{X}}_{\text{9q}}}{\text{7}}\right)+{\text{X}}_{\text{10}}] \end{array}$$ (2)

### 3.3. Construction of the PMC surface plot

The PMC surface is generated using the PMC index to visualize the policy evaluation results. This graphical representation offers an intuitive way of highlighting the disparities across various policy indicators. Presenting the information in a three-dimensional and highly visual manner, facilitates a direct comparison of the strengths and weaknesses of various policies, thus enabling scholars to conduct comprehensive policy analysis. In this study, 10 primary variables were initially defined. However, the variable related to policy disclosure (X10) was excluded due to its insignificant contributions to the overall policy evaluation, maintaining the balance and symmetry of the PMC surface [22,24,26]. Subsequently, the remaining nine primary variables, denoted as X1 through X9, were utilized to construct a 3 × 3 surface matrix following Formula (3). Based on this matrix, the PMC surface plot was then constructed.


$$\begin{array}{c} \text{PMC}-\text{Surface}=[\begin{array}{ccc} \text{X1} & \text{X2} & \text{X3}\\ \text{X4} & \text{X5} & \text{X6}\\ \text{X7} & \text{X8} & \text{X9} \end{array}] \end{array}$$ (3)

## 4. Results

### 4.1 PMC index value

The PMC index values for the 18 policies were calculated using Formulas (1) and (2) and then classified according to the aforementioned classification criteria. As indicated in Table 3, the mean PMC index value of the 18 policies is 6.28, indicating an overall policy performance level of “Good.” The ranking of the policies is as follows: P11 > P6 > P15 > P9 > P12 > P16 > P10 > P5 > P14 > P2 > P7 > P4 > P1 = P17 > P13 > P3 > P18 > P8. Specifically, six policies were rated as “Excellent,” rating, twelve were rated as “Good,” and none were classified as either “Perfect” or “Unqualified.”

**Table 3:** PMC index value and policy level evaluation table of 19 policies.

Code	X1	X2	X3	X4	X5		X6
P1	0.67	0.50	0.20	0.25	0.80		0.67
P2	0.67	0.50	0.20	0.25	0.80		0.67
P3	0.67	0.50	0.20	0.25	0.60		0.67
P4	0.67	0.50	0.20	0.25	0.40		1.00
P5	0.67	0.50	0.20	0.25	0.60		1.00
P6	1.00	0.50	0.20	0.25	0.60		1.00
P7	1.00	0.50	0.20	0.25	0.60		0.67
P8	0.67	0.50	0.20	0.25	0.60		0.67
P9	1.00	0.50	0.20	0.25	0.60		1.00
P10	0.67	0.50	0.20	0.25	1.00		0.67
P11	1.00	0.50	0.20	0.25	0.60		1.00
P12	1.00	0.50	0.20	0.25	1.00		0.67
P13	1.00	0.50	0.20	0.25	0.60		0.67
P14	1.00	0.50	0.20	0.25	1.00		0.67
P15	0.67	0.50	0.20	0.25	0.60		0.67
P16	0.67	0.50	0.20	0.25	0.80		1.00
P17	0.67	0.50	0.20	0.25	0.60		0.67
P18	0.67	0.50	0.20	0.25	0.60		0.67
average	0.80	0.50	0.20	0.25	0.69		0.78
Code	X7	X8	X9	X10	PMC Index	degree	rank
P1	0.33	1.00	0.43	1.00	5.85	Good	13
P2	0.33	1.00	0.71	1.00	6.13	Good	10
P3	0.33	1.00	0.57	1.00	5.79	Good	16
P4	0.67	0.50	0.71	1.00	5.90	Good	12
P5	1.00	0.50	0.71	1.00	6.43	Good	8
P6	0.67	1.00	1.00	1.00	7.22	Excellent	2
P7	0.33	1.00	0.43	1.00	5.98	Good	11
P8	0.33	0.50	0.71	1.00	5.43	Good	18
P9	0.33	1.00	0.86	1.00	6.74	Excellent	4
P10	0.33	1.00	0.86	1.00	6.48	Good	7
P11	1.00	1.00	0.86	1.00	7.41	Excellent	1
P12	0.67	1.00	0.43	1.00	6.72	Excellent	5
P13	0.33	1.00	0.29	1.00	5.84	Good	15
P14	0.33	1.00	0.43	1.00	6.38	Good	9
P15	1.00	1.00	0.86	1.00	6.75	Excellent	3
P16	1.00	0.50	0.71	1.00	6.63	Excellent	6
P17	0.67	1.00	0.29	1.00	5.85	Good	13
P18	1.00	0.50	0.14	1.00	5.53	Good	17
average	0.59	0.86	0.61	1.00	6.28	Good	

### 4.2. Horizontal analysis

Aiming to comprehensively assess the overall efficacy of policies governing the online sale of prescription drugs, a multi-dimensional horizontal analysis of 18 relevant policies was conducted. The average score for dimension X1 across the 18 policies is 0.80. While all policies exhibit either regulatory or advisory characteristics, only a limited number integrate both aspects simultaneously. The lack of detailed and systematic guidelines within these policies has led to substantial implementation challenges, thereby impeding the effective execution of regulations governing the online sale of prescription drugs. Dimensions X2, X3, X4, and X10 serve as single-criterion evaluation indicators. Notably, X2 has an average score of 0.5. Eight policies (P1, P2, P3, P4, P5, P8, P9, and P10) impose outright bans on the online sale of prescription drugs, whereas the remaining ten policies adopt more relaxed these restrictions. This disparity highlights the historical uncertainty and the current trend toward cautious liberalization in China’s regulatory approach to the online sale of prescription drugs. With an average score of 0.2, dimension X3 reveals that the majority of policies are unilaterally issued by drug regulatory authorities, highlighting notable limitations in inter-departmental collaboration. This compartmentalized, department-specific approach undermines the construction of a cohesive regulatory framework. The average score of 0.25 for dimension X4 is indicative of the legislative landscape, which comprises 12 departmental regulations, 3 departmental working documents, 2 administrative regulations, and a single legal document. This distribution highlights the relatively low legislative priority given to policies governing the online sale of prescription drugs in China. Legal Document P10 clarifies the legality of the online sale of prescription drugs at the statutory level. Subsequently, supporting administrative regulations and departmental rules have been successively issued, and in strict accordance with the relevant provisions of the Pharmaceutical Administration Law, targeted measures have been implemented to regulate key areas such as the division of responsibilities, supervision standards, and penalty rules governing the online sale of prescription drugs. This policy evolution fully reflects the guiding and dominant position of superior legal documents, centered on the Pharmaceutical Administration Law governing the online sale of prescription drugs. With an average score of 0.69, dimension X5 indicates that current policies attach considerable importance to regulating relevant entities involved in the online sale of prescription drugs. However, during the weight assignment process, among the policies explicitly permitting the online sale of prescription drugs, only four (P10, P12, P15, and P16) simultaneously involve medical institutions and third-party platforms. Moreover, the relevant provisions failed to clarify the boundaries of rights and obligations between the two parties in relation to the online sale of prescription drugs. Therefore, future policy development should focus on refining the division of responsibilities between medical institutions and third-party platforms, regulating the behaviors of the two types of entities in relation to the online sale of prescription drugs, and enhancing the pertinence and enforceability of the policies. An average score of 0.78 for dimension X6 indicates that certain policies effectively combine functions of supervision, restraint, standardization, and guidance, while also offering practical recommendations, demonstrating a comprehensive use of policy instruments. With an average score of 0.59, dimension X7 highlights that approximately half of the policies allocate less than 5% of their content to the online sale of prescription drugs, reflecting historical oversight. This oversight indicates a need for increased attention on this area in future policy development. The high average score of 0.86 for dimension X8 demonstrates that policies adopt macro-and micro-level perspectives to systematically plan the online sale of prescription drugs, regulate online drug transactions, and oversee platform services, thereby safeguarding public health and medication safety. Finally, with an average score of 0.61, dimension X9 shows that while policies generally cover key areas such as access control, punitive measures, transaction monitoring, and information management, only a limited number of policies address aspects such as quality control, advertising regulation, and distribution supervision. This limitation exposes considerable gaps in China’s regulatory system in relation to the online sale of prescription drugs, emphasizing the necessity for continuous refinement and expansion.

### 4.3. Vertical analysis

**Fig 2:**
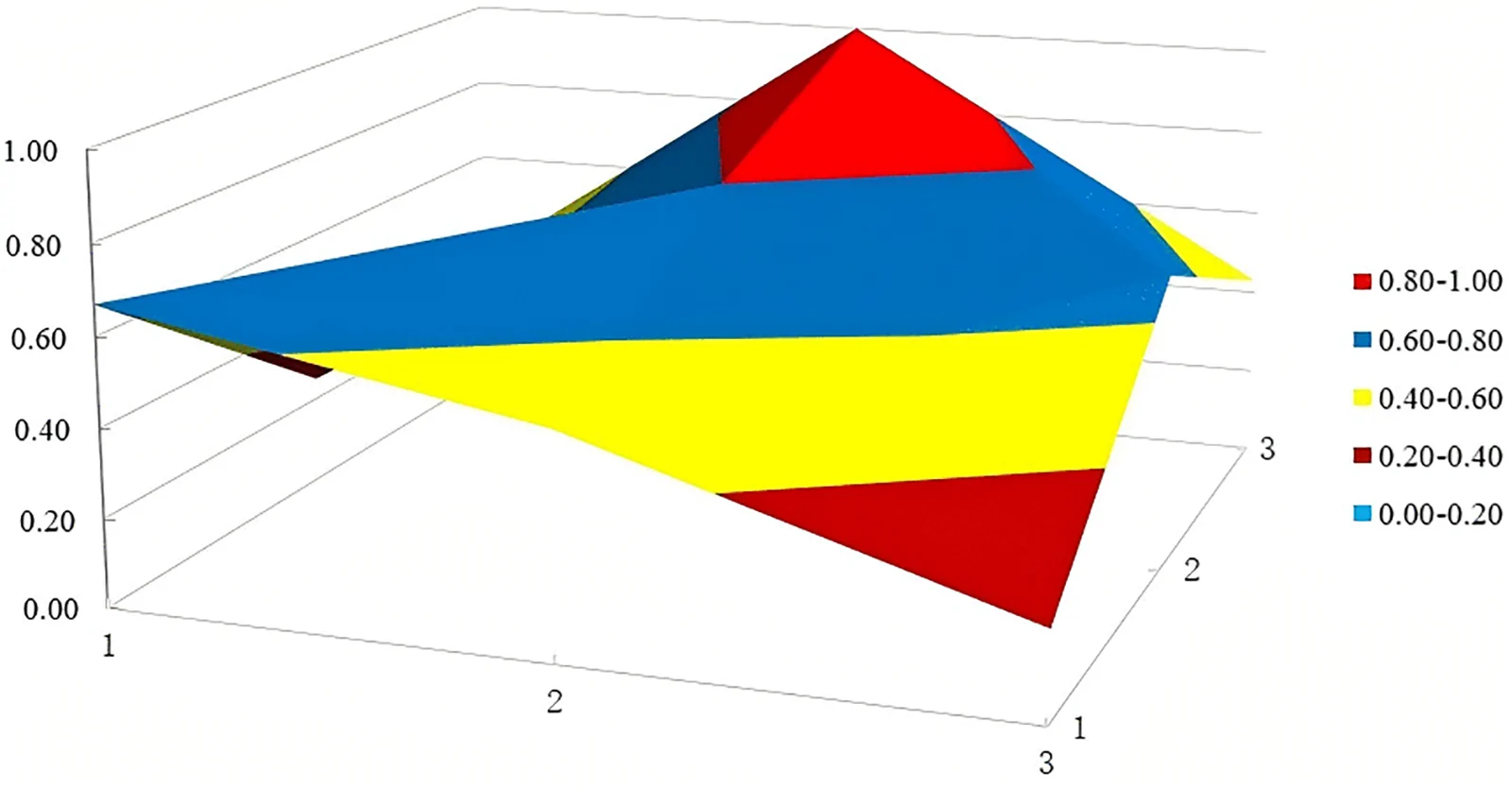
PMC-Surface for P1.

**Fig 3:**
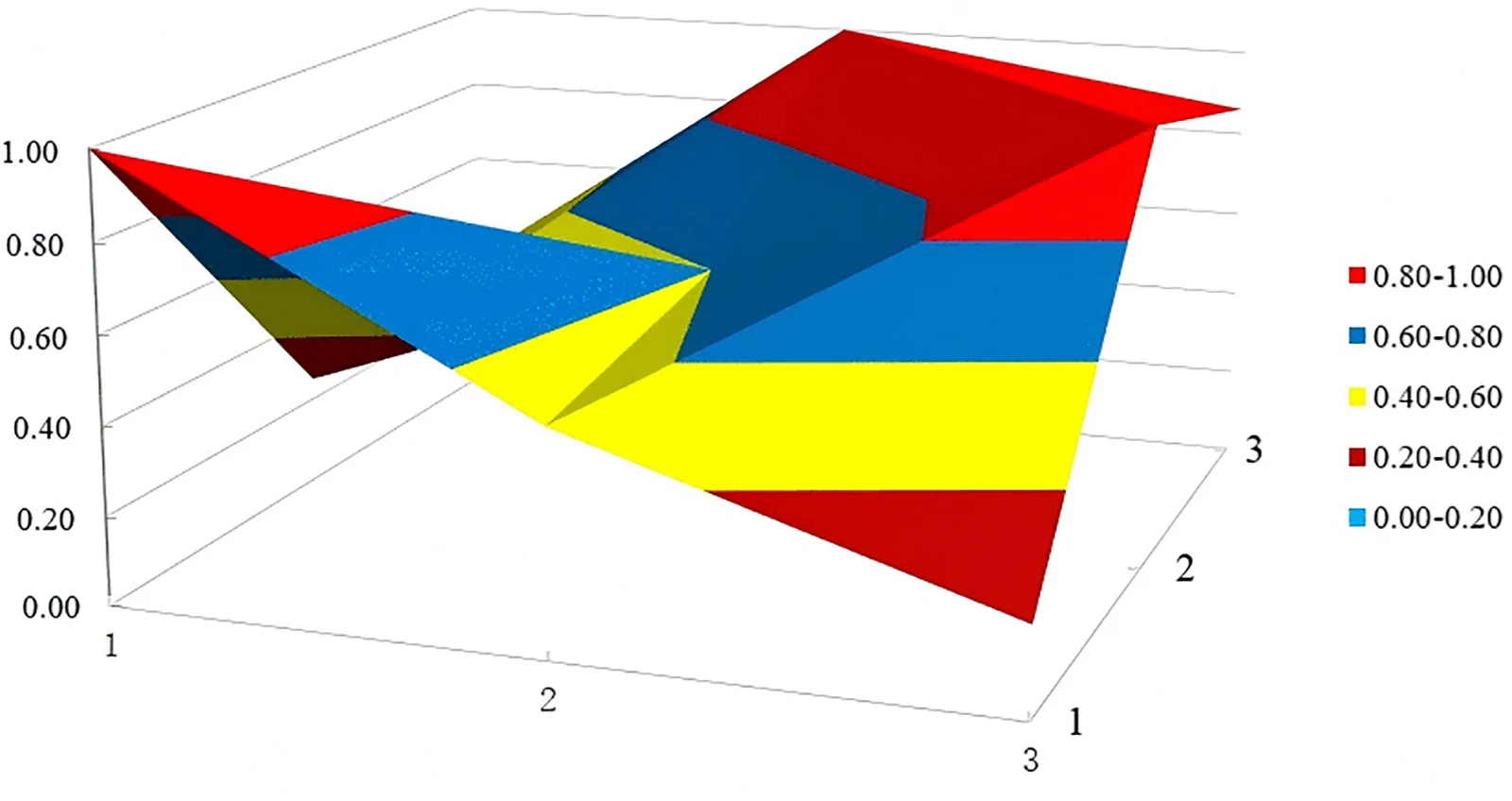
PMC-Surface for P9.

**Fig 4:**
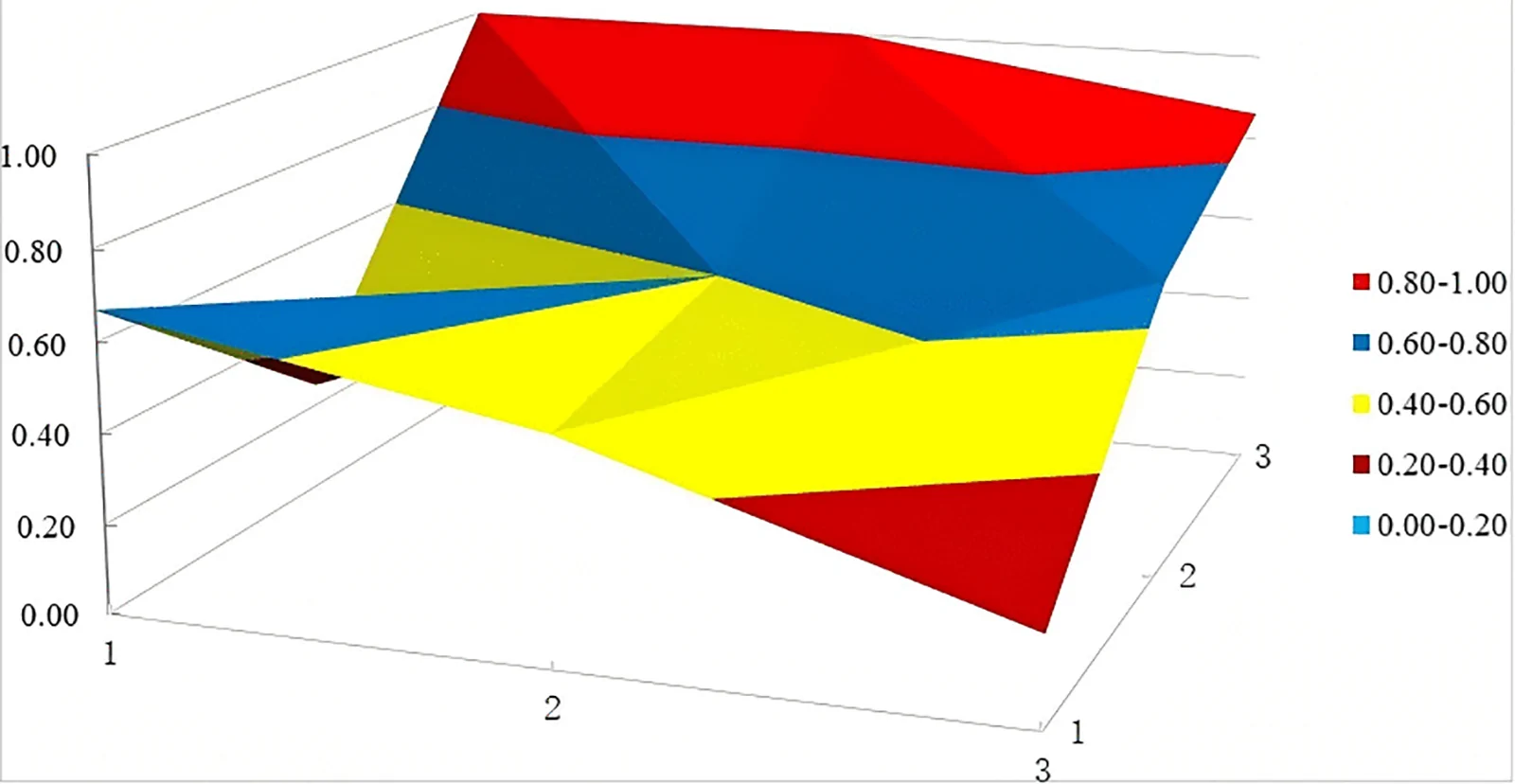
PMC-Surface for P15.

**Fig 5:**
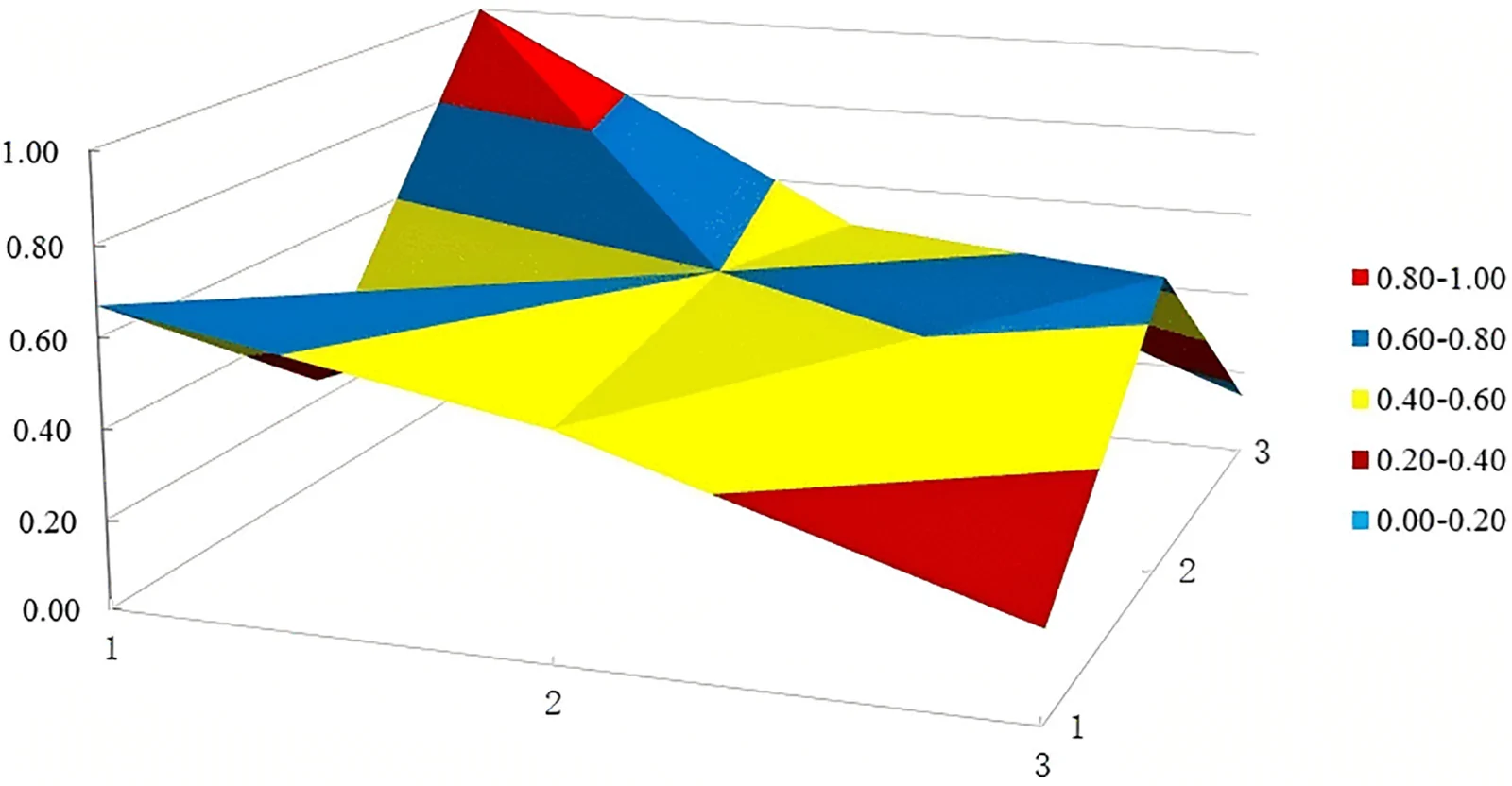
PMC-Surface for P18.

Vertical analysis provides a highly intuitive means of exposing the disparities and extant issues among different policies. In this study, based on the policies that prohibited the online sale of prescription drugs, the highest-scoring policy (P9, Excellent) and a lower-scoring policy (P1, Good) were selected for comparison. Similarly, from the policies that permitted such sales, P15 (Excellent) and P18 (Good) were chosen. Based on the four selected policies, a PMC surface graph was constructed (Fig 2, Fig 3, Fig 4, Fig 5) to conduct a specific analysis of the advantages and challenges governing the online sale of prescription drugs.

The PMC index for P1 is 5.85, whereas that of P9 reaches 6.74, indicating a notable disparity of 0.89 between the two policies. As shown in Table 3, P1, as the pioneering regulatory policy prohibiting the online sale of prescription drugs, exhibits inherent limitations in addressing critical dimensions such as X1 policy nature, X6 policy function, and X9 policy content. Consequently, its scores across these parameters are substantially lower than those of P9. The subsequent introduction of P9 effectively rectifies several regulatory deficiencies identified in P1. However, P9 still fails to establish a robust framework for regulating prescription drug advertisements, highlighting an area that warrants further policy refinement.

The PMC index for P15 is 6.75, whereas that of P18 reaches 5.53, revealing a substantial 1.22-point disparity between the two policies. Notably, P18 demonstrates lower scores than P15 across critical dimensions, particularly in X8 policy perspective and X9 policy content. Although P18 serves as an extension aimed at operationalizing the provisions of P15, its scope remains confined predominantly to regulating the information disclosure aspects in relation to the online sale of prescription drugs. Importantly, this policy omits crucial regulatory domains such as market access criteria, quality control mechanisms, and distribution management systems integral to the online sale of prescription drugs. Compared with its antecedent policies, P15 represents a notable advancement in prioritizing the online sale of prescription drugs, as evidenced by the elevated proportion of policy text dedicated to this domain. However, a clear gap persists in its regulatory framework, as P15 fails to establish comprehensive guidelines for advertisement supervision. This gap highlights the need for future policy revisions to incorporate robust mechanisms for regulating prescription drug advertising, thereby addressing a critical aspect of the regulatory framework governing online pharmaceutical commerce.

Additionally, based on the PMC policy evaluation index system, this study calculated the primary variable values and PMC index of the U.S. Federal Food, Drug, and Cosmetic Act, excluding four single-criterion evaluation primary variables: X2, X3, X4, and X10. The values of the remaining six core primary variables are as follows: X1 is 0.67, while X5, X6, X7, X8, and X9 each have a value of 1. Compared with Policy P11, which is equivalent in legislative hierarchy in China, the US policy lags behind only in two primary variables: X5 and X9. Future policy optimization should focus on addressing regulatory loopholes in the supervision domain and further strengthening standardized constraints on medical institutions and social organizations as participating entities.

### 4.4. Indicator weight analysis

Aiming to identify the importance of indicators in the evaluation system for policies governing the online sale of prescription drugs, the entropy weight method was used in this study to calculate objective weights. Four primary indicators (X2, X3, X4, X10) yield identical values with no distinguishable differences in importance for weighting; therefore, they are excluded, and weight calculations were performed only for the remaining six indicators. The original data of the indicator components were normalized in accordance with Equation (4). Subsequently, the information entropy was calculated using Equations (5) and (6), the difference coefficient was computed via Equation (7), and the weights of each indicator component were determined using Equation (8). The results are presented in Table 4, where m denotes the number of evaluated objects, and n represents the number of evaluation indicators.

**Table 4:** Summary of weight calculation results via the entropy method.

Indicator	X1	X5	X6	X7	X8	X9
Entropy value	0.703042	0.943769	0.657695	0.766373	0.894784	0.948523
Coefficient of Variation	0.296957	0.056231	0.342305	0.233627	0.105216	0.051477
Weight	0.273488	0.051787	0.315252	0.215163	0.096901	0.047409


$$\begin{array}{c} x_{ij}=\frac{x_{ij}-\text{min}(x_{j})}{\text{max}\left(x_{j}\right)-\text{min}(x_{j})} \end{array}$$ (4)

$$\begin{array}{c} p_{ij}=\frac{x_{ij}^{n}}{\sum_{i=\text{1}}^{n}x_{ij}^{n}} \end{array}$$ (5)

$$\begin{array}{c} e_{j}=-\text{ln}n\sum_{i=\text{1}}^{n}p_{ij}\text{ln}p_{ij} \end{array}$$ (6)

$$\begin{array}{c} g_{j}=\text{1}-e_{j} \end{array}$$ (7)

$$\begin{array}{c} w_{j}=\frac{g_{j}}{\sum_{k=\text{1}}^{m}g_{k}} \end{array}$$ (8)

The results show that indicator X6 has the highest weight, implying that current policies governing the online sale of prescription drugs prioritize macro-level guidance and regulation. By clarifying the boundaries of industry development and standardizing the conduct of relevant stakeholders, these policies effectively define the core direction of regulatory supervision. Conversely, X9 registers the lowest weight, indicating that China has yet to establish a comprehensive full-process closed-loop regulatory system for the online sale of prescription drugs. Weak linkages between different regulatory segments and inefficient cross-departmental coordination further limit the effectiveness of governance. Overall, these findings verify that current policies prioritize macro-level guidance while adopting refined regulation as a supplementary measure.

Future policies should be jointly formulated by the National Medical Products Administration, the Ministry of Industry and Information Technology, the State Post Bureau, and other relevant authorities. Regulators should clearly define full-process regulatory responsibilities related to the online sale of prescription drugs, strengthen cross-agency coordination mechanisms, and improve the comprehensive closed-loop supervision system across the entire industry chain.

### 4.5. LDA model analysis

In this paper, the Latent Dirichlet Allocation (LDA) model is employed to conduct topic mining on policies governing the online sale of prescription drugs. The optimal number of topics was determined using two evaluation metrics: the perplexity curve and the topic coherence score. The model achieved an overall average topic coherence score of 0.46. This value falls within a reasonable range due to the limited sample size of policy texts in this study. Combined with the global minimum perplexity value and the semantic interpretability of feature keywords for each topic, three was ultimately selected as the optimal number of topics. Based on the feature words associated with each topic, the three themes were summarized as qualification supervision of online pharmaceutical sales, regulation of the online sale of prescription drugs, and the construction of a digital-intelligent regulatory system, as shown in Table 5. The table reveals that the qualification supervision of online pharmaceutical sales carries the highest weight, followed by the construction of a digital-intelligent regulatory system, while regulation of the online sale of prescription drugs has the lowest weight. This result indicates that current Chinese policies are relatively well-established in terms of access qualification for online pharmaceutical sales, but remain inadequate in terms of whole-process regulation of the online sale of prescription drugs. This finding is highly consistent with the conclusion from Dimension X9 in Section 4.4 which indicates that a comprehensive closed-loop regulatory system for the online sale of prescription drugs has not yet been fully established, thereby verifying the robustness of the results of the PMC index model. Meanwhile, the Chinese government has increasingly recognized the advantages of smart regulation and is continuously advancing the development of a smart regulatory system. Future policies should further clarify the regulatory responsibilities at each stage of the process and address weaknesses in whole-process governance. Additionally, efforts should be accelerated to establish an integrated digital-intelligent regulatory system, thus forming a highly efficient regulatory framework featuring cross-departmental collaboration, full-chain coverage, and smart governance.

**Table 5:** Topic-feature words of the 18 policy texts.

Theme	Feature Words	Weight/%	Coherence Score
Qualification Supervision of Online Pharmaceutical Sales	Internet, Pharmaceuticals, Websites, Operators, Consumers, Filing, Qualification Certificates, Laws	44.6	0.41
Control of Online Prescription Drug Sales	Platforms, Fines, Medical Institutions, Inspections, Prescriptions, Violations, Sellers, Supervision	27.2	0.42
Construction of Smart Regulatory System	Internet, Reform, Fields, Platforms, Construction, Data, Technology, Supervision, Optimization, Industry, Institutions, System	28.2	0.54

## 5. Discussion

Three of the six excellent policies are drafts that were released for public comment, indicating that the regulation of the online sale of prescription drugs in China is still in the exploratory stage. The current policy framework is not comprehensive, and several implementation challenges are encountered, including the lack of detailed rules for regulating various subjects, the cross-functional responsibilities of various departments with unclear boundaries between rights and responsibilities, and the urgent need to improve digital supervision capabilities.

The score of “Policy Targets (X5)” is 0.69, which is relatively higher than that of other first-level indicators. However, among the 18 policies, only seven mention medical institutions. Mechanisms such as corresponding diagnostic and treatment standards, the legitimacy of prescription-issuing entities, and the protection of medical information and patient privacy remain underdeveloped [27]. Moreover, due to regional policy variations, unified standards for service specifications, including pharmaceutical care, electronic medical records, and online prescriptions, are currently lacking, thereby posing considerable obstacles to policy implementation [28–30]. Subsequent policies should regulate the behaviors of Internet hospitals and further refine online diagnosis and treatment procedures. They should also unify electronic prescription standards to ensure the smooth circulation of electronic prescriptions and establish robust information security systems for Internet hospitals to protect patient privacy [31,32].

The score for “Policy Importance (X7)” is 0.59, which is relatively low. This score may be attributed to the complicated liberalization process of online sale of prescription drugs in China and the absence of a sound legal and regulatory system. Therefore, subsequent policies should be refined and standardized in the following aspects: strictly reviewing the access qualifications of pharmaceutical enterprises and establishing a multi-dimensional access mechanism covering business licenses, quality management system certification, and Internet pharmaceutical information service qualifications. Prescription circulation should be standardized by constructing an integrated closed-loop management system covering the entire process of “issuing–reviewing– circulating–using–archiving,” thereby creating a healthy prescription circulation environment. Additionally, pharmacist services should be regulated by introducing corresponding standard guidelines for prescription review and medication guidance. After-sales services should also be improved, requiring third-party platforms and merchants to refine adverse drug reaction monitoring and feedback system and implement consumer rights protection [33].

The score for “Policy Content (X9)” is 0.61. Most policies lack detailed regulations on quality supervision, distribution supervision, and advertising regulation, leading to issues such as overlapping departmental responsibilities, unclear division of duties, and low regulatory efficiency. Quality supervision, as a core mechanism for ensuring drug quality, is indispensable. Therefore, the government should delegate responsibilities, refine the duties of each department, establish a reasonable reward and punishment mechanism, hold the primary negligent departments accountable, and ensure the quality and safety of prescription drugs [34]. The distribution process represents a critical node where drug quality risks are prone to emerge. In the future, raising the threshold for prescription drug logistics by establishing corresponding standards for personnel qualifications, transportation vehicles, and other aspects is necessary. Regular supervisory inspections of logistics platforms should be performed, while a full-cycle traceability system for the logistics and distribution of prescription drugs should also be developed [35]. Advertising is the primary channel through which consumers learn information about pharmaceuticals; therefore, false or misleading information in advertisements poses notable risks to consumer safety [36–38]. Subsequent policies should improve the regulatory system for prescription drug advertising by improving the review system for prescription drug advertisements and establishing management norms for advertising and pharmaceutical marketing [39,40]. Additionally, multiple departments should collaborate efforts with industry associations to establish an intelligent regulatory team comprising professional talents from multiple fields, including pharmacy, information technology, and law. This strategy would promote the formation of a three-dimensional digital regulatory framework.

In addition, the sensitivity analysis of all primary variables reveals that X3 and X4 exert a relatively substantial impact on overall policy effectiveness. Notably, when the value of X3 is increased, the PMC index of the optimal policy reaches a maximum of 8.21, which is only 0.29 lower than that of an ideal policy (8.50). Similarly, when the value of X4 is elevated, the PMC index peaks at 8.16, representing a gap of 0.34 from the ideal policy. This demonstrates that optimizing the values of X3 and X4 is highly meaningful for policy improvement. However, owing to the fixed nature of policy legal effect and hierarchy, the value of X4 is inherently rigid. Therefore, future policy efforts should focus on optimizing variable X3 by supporting joint policy formulation across multiple departments, improving cross-departmental collaborative governance mechanisms, and enhancing the overall systematicity and coordination of the policy system.

## 6. Limitation

Local-level policies have not been fully enacted; thus, this study only includes national-level policy samples. Future research could incorporate local policies and perform comparative analyses between national and local policies. Owing to the limited number of policy samples, the variables selected in this paper based on text mining have certain limitations, and some degree of subjectivity remains in variable selection. In subsequent research, the dimensions and extensions of the variables may be further expanded to improve the robustness of the quantitative evaluation index system for policies governing the online sale of prescription drugs. For example, the synergistic effect could be used to explore the connections between various policies, providing a reference basis for the development of scientific and rigorous policies in the future.

## Supporting information

S1 Supplementary Charts and Figures Supplementary materials contain the basis for computing PMC-index values as well as PMC surface diagrams for additional policies. (DOCX)

S1 Row Feature Words – Co-word Matrix This dataset serves as the data foundation for constructing the keyword social network graph for online prescription-drug sales policies. (XLSX)
