# Supplementary material for: Quantitative assessment of china’s policies on the online sale of prescription drugs
Source: PLoS One. 2026 Sep 23;21(9):e0358484. doi: 10.1371/journal.pone.0358484 (PMC13600529; doi:10.1371/journal.pone.0358484)
Supplement: Supplementary file 1 [file pone.0358484.s001.docx]

Multi-Input-Output Table.

| Code | X1 |  |  | X2 |  | X3 |  |  |  |
| --- | --- | --- | --- | --- | --- | --- | --- | --- | --- |
|  | X1:1 | X1:2 | X1:3 | X2:1 | X2:2 | X3:1 | X3:2 | X3:3 | X3:4 |
| P1 | 1 | 1 | 0 | 1 | 0 | 1 | 0 | 0 | 0 |
| P2 | 1 | 1 | 0 | 1 | 0 | 1 | 0 | 0 | 0 |
| P3 | 1 | 1 | 0 | 1 | 0 | 1 | 0 | 0 | 0 |
| P4 | 0 | 1 | 1 | 1 | 0 | 1 | 0 | 0 | 0 |
| P5 | 0 | 1 | 1 | 1 | 0 | 1 | 0 | 0 | 0 |
| P6 | 1 | 1 | 1 | 0 | 1 | 1 | 0 | 0 | 0 |
| P7 | 1 | 1 | 1 | 0 | 1 | 0 | 0 | 1 | 0 |
| P8 | 1 | 1 | 0 | 1 | 0 | 1 | 0 | 0 | 0 |
| P9 | 1 | 1 | 1 | 1 | 0 | 1 | 0 | 0 | 0 |
| P10 | 1 | 1 | 0 | 1 | 0 | 0 | 0 | 0 | 1 |
| P11 | 1 | 1 | 1 | 0 | 1 | 1 | 0 | 0 | 0 |
| P12 | 1 | 1 | 1 | 0 | 1 | 0 | 0 | 0 | 0 |
| P13 | 1 | 1 | 1 | 0 | 1 | 0 | 0 | 1 | 0 |
| P14 | 1 | 1 | 1 | 0 | 1 | 0 | 0 | 0 | 0 |
| P15 | 1 | 1 | 0 | 0 | 1 | 0 | 1 | 0 | 0 |
| P16 | 0 | 1 | 1 | 0 | 1 | 1 | 0 | 0 | 0 |
| P17 | 1 | 1 | 0 | 0 | 1 | 0 | 1 | 0 | 0 |
| P18 | 1 | 1 | 0 | 0 | 1 | 1 | 0 | 0 | 0 |

Continue

| Code | X3 | X4 |  |  |  | X5 |  |  |  |
| --- | --- | --- | --- | --- | --- | --- | --- | --- | --- |
|  | X3:5 | X4:1 | X4:2 | X4:3 | X4:4 | X5:1 | X5:2 | X5:3 | X5:4 |
| P1 | 0 | 0 | 0 | 1 | 0 | 1 | 1 | 1 | 0 |
| P2 | 0 | 0 | 0 | 1 | 0 | 1 | 1 | 1 | 1 |
| P3 | 0 | 0 | 0 | 1 | 0 | 1 | 1 | 1 | 0 |
| P4 | 0 | 0 | 0 | 1 | 0 | 1 | 0 | 0 | 1 |
| P5 | 0 | 0 | 0 | 1 | 0 | 1 | 1 | 0 | 1 |
| P6 | 0 | 0 | 0 | 1 | 0 | 1 | 1 | 0 | 1 |
| P7 | 0 | 0 | 1 | 0 | 0 | 1 | 1 | 0 | 0 |
| P8 | 0 | 0 | 0 | 0 | 1 | 1 | 1 | 0 | 0 |
| P9 | 0 | 0 | 0 | 1 | 0 | 1 | 1 | 0 | 1 |
| P10 | 0 | 1 | 0 | 0 | 0 | 1 | 1 | 1 | 1 |
| P11 | 0 | 0 | 0 | 1 | 0 | 1 | 1 | 0 | 1 |
| P12 | 1 | 0 | 0 | 1 | 0 | 1 | 1 | 1 | 1 |
| P13 | 0 | 0 | 1 | 0 | 0 | 1 | 1 | 0 | 0 |
| P14 | 1 | 0 | 0 | 1 | 0 | 1 | 1 | 1 | 1 |
| P15 | 0 | 0 | 0 | 1 | 0 | 1 | 1 | 0 | 1 |
| P16 | 0 | 0 | 0 | 0 | 1 | 1 | 1 | 1 | 1 |
| P17 | 0 | 0 | 0 | 1 | 0 | 1 | 1 | 0 | 0 |
| P18 | 0 | 0 | 0 | 0 | 1 | 1 | 1 | 0 | 1 |

Continue

| Code | X5 | X6 |  |  | X7 |  |  | X8 |  |
| --- | --- | --- | --- | --- | --- | --- | --- | --- | --- |
|  | X5:5 | X6:1 | X6:2 | X6:3 | X7:1 | X7:2 | X7:3 | X8:1 | X8:2 |
| P1 | 1 | 1 | 1 | 0 | 1 | 0 | 0 | 1 | 1 |
| P2 | 0 | 1 | 1 | 0 | 1 | 0 | 0 | 1 | 1 |
| P3 | 0 | 1 | 1 | 0 | 1 | 0 | 0 | 1 | 1 |
| P4 | 0 | 1 | 1 | 1 | 1 | 1 | 0 | 0 | 1 |
| P5 | 0 | 1 | 1 | 1 | 1 | 1 | 1 | 0 | 1 |
| P6 | 0 | 1 | 1 | 1 | 1 | 1 | 0 | 1 | 1 |
| P7 | 1 | 0 | 1 | 1 | 1 | 0 | 0 | 1 | 1 |
| P8 | 1 | 1 | 1 | 0 | 1 | 0 | 0 | 0 | 1 |
| P9 | 0 | 1 | 1 | 1 | 1 | 0 | 0 | 1 | 1 |
| P10 | 1 | 1 | 1 | 0 | 1 | 0 | 0 | 1 | 1 |
| P11 | 0 | 1 | 1 | 1 | 1 | 1 | 1 | 1 | 1 |
| P12 | 1 | 0 | 1 | 1 | 1 | 1 | 0 | 1 | 1 |
| P13 | 1 | 0 | 1 | 1 | 1 | 0 | 0 | 1 | 1 |
| P14 | 1 | 0 | 1 | 1 | 1 | 0 | 0 | 1 | 1 |
| P15 | 0 | 1 | 1 | 0 | 1 | 1 | 1 | 1 | 1 |
| P16 | 0 | 1 | 1 | 1 | 1 | 1 | 1 | 0 | 1 |
| P17 | 1 | 1 | 1 | 0 | 1 | 1 | 0 | 1 | 1 |
| P18 | 0 | 1 | 1 | 0 | 1 | 1 | 1 | 0 | 1 |

Continue

| Code | X9 |  |  |  |  |  |  | X10 |
| --- | --- | --- | --- | --- | --- | --- | --- | --- |
|  | X9:1 | X9:2 | X9:3 | X9:4 | X9:5 | X9:6 | X9:7 | X10:1 |
| P1 | 1 | 1 | 0 | 1 | 0 | 0 | 0 | 1 |
| P2 | 1 | 0 | 1 | 1 | 0 | 1 | 1 | 1 |
| P3 | 1 | 0 | 1 | 1 | 0 | 1 | 0 | 1 |
| P4 | 1 | 0 | 1 | 1 | 1 | 0 | 1 | 1 |
| P5 | 1 | 0 | 1 | 1 | 0 | 1 | 1 | 1 |
| P6 | 1 | 1 | 1 | 1 | 1 | 1 | 1 | 1 |
| P7 | 0 | 1 | 0 | 1 | 0 | 0 | 1 | 1 |
| P8 | 1 | 1 | 0 | 1 | 0 | 1 | 1 | 1 |
| P9 | 1 | 1 | 1 | 1 | 0 | 1 | 1 | 1 |
| P10 | 1 | 1 | 1 | 1 | 1 | 0 | 1 | 1 |
| P11 | 1 | 1 | 1 | 1 | 0 | 1 | 1 | 1 |
| P12 | 1 | 0 | 0 | 1 | 0 | 0 | 1 | 1 |
| P13 | 1 | 0 | 0 | 1 | 0 | 0 | 0 | 1 |
| P14 | 1 | 0 | 0 | 1 | 0 | 0 | 1 | 1 |
| P15 | 1 | 1 | 1 | 1 | 0 | 1 | 1 | 1 |
| P16 | 1 | 1 | 0 | 1 | 0 | 1 | 1 | 1 |
| P17 | 0 | 0 | 1 | 0 | 1 | 0 | 0 | 1 |
| P18 | 0 | 0 | 0 | 0 | 0 | 0 | 1 | 1 |

PMC surface diagram for policy P2.

PMC surface diagram for policy P3.

PMC surface diagram for policy P4.

PMC surface diagram for policy P5.

PMC surface diagram for policy P6.

PMC surface diagram for policy P7.

PMC surface diagram for policy P8.

PMC surface diagram for policy P10.

PMC surface diagram for policy P11.

PMC surface diagram for policy P12.

PMC surface diagram for policy P13.

PMC surface diagram for policy P14.

PMC surface diagram for policy P16.

PMC surface diagram for policy P17.

PMC Surface Diagrams of 18 Policies.

Radar Chart of First-level Indicators for 18 Policies.

Radar Chart of First-level Indicators for Policies P1 and P9.

Radar Chart of First - level Indicators for Policies P15 and P18.
