# Supplementary material for: Quantitative assessment of china’s policies on the online sale of prescription drugs
Source: PLoS One. 2026 Sep 23;21(9):e0358484. doi: 10.1371/journal.pone.0358484 (PMC13600529; doi:10.1371/journal.pone.0358484)
Supplement: Supplementary file 2 [file pone.0358484.s003.docx]

**QUANTITATIVE ASSESSMENT OF CHINA’S POLICIES ON THE NETWORK SALES OF PRESCRIPTION DRUGS BASED ON THE PMC INDEX MODEL: REVIEWER COMMENTS**

**General Comments**

This study addresses a critical and timely issue by quantitatively evaluating China's regulatory policies on the online sales of prescription drugs through a structured PMC Index Model combined with text mining techniques. In light of the rapid expansion of digital healthcare platforms and the associated public safety risks, the authors' systematic assessment of policy consistency and regulatory comprehensiveness is highly valuable. The integration of policy text analysis with quantitative modeling offers a clear and replicable framework for policy evaluation, and the findings provide meaningful insights into both the progress and existing gaps within China's evolving regulatory system. Overall, the research makes a significant contribution to the literature on digital health governance and public policy evaluation.

**Recommendations for Improvement**

To further enhance the rigor, analytical depth, and policy relevance of the study, the following expert recommendations are proposed:

1. **Apply Differential Variable Weighting**

The current binary scoring system assigns equal importance to all variables. The authors should incorporate weighting methods such as the Analytic Hierarchy Process (AHP) or the Entropy Weight Method to reflect the varying importance of regulatory dimensions, particularly prioritizing critical aspects such as quality supervision and public safety.

2. **Perform a Policy Conflict and Legal Hierarchy Analysis**

Given that most policies are departmental regulations with relatively low legislative priority, the authors should analyze potential inconsistencies or conflicts between these documents and higher-level legislation, particularly the Medicinal Product Administration Law. This would provide deeper legal and institutional insights.

3. **Integrate Advanced Topic Modelling Techniques (e.g., LDA)**

While keyword frequency and network analysis are informative, employing Latent Dirichlet Allocation (LDA) would allow for the identification of latent thematic structures and policy evolution trends, offering a more sophisticated understanding of regulatory focus over time.

4. **Refine and Expand the “Policy Target” (X5) Dimension**

The evaluation of policy objects should be further subdivided to distinguish between regulatory obligations, rights, and accountability mechanisms for medical institutions and third-party platforms. This would help clarify governance responsibilities and enhance analytical precision.

5. **Conduct International Benchmarking Comparisons**

The study would benefit from applying the PMC framework to selected international regulatory systems (e.g., U.S. or EU online pharmacy regulations). Such benchmarking would contextualize China’s policy performance and identify global best practices.

6. **Develop a Predictive Policy Roadmap through Sensitivity Analysis**

By simulating a “perfect” policy scenario and analyzing which variables most significantly influence the PMC Index, the authors could provide actionable guidance on priority areas for future regulatory reform.

**Concluding Statement**

This is a promising and well-structured study that offers valuable quantitative insights into the regulation of online prescription drug sales in China. With the incorporation of the recommended methodological enhancements and deeper analytical components, the manuscript has strong potential to make a substantial contribution to policy evaluation research. I recommend that the study be accepted for publication following satisfactory revision addressing the above points.
