# Supplementary material for: Quantitative assessment of china’s policies on the online sale of prescription drugs
Source: PLoS One. 2026 Sep 23;21(9):e0358484. doi: 10.1371/journal.pone.0358484 (PMC13600529; doi:10.1371/journal.pone.0358484)
Supplement: Supplementary file 3 [file pone.0358484.s004.docx]

***Summary of major changes:***

*<1> In accordance with the experts' suggestions, add a new chapter to explore the weight of primary indicators by using the entropy weight method, and analyze their paths and implications for policy optimization.*

*<2> Analyze the binding force of superior legislation such as the "Drug Administration Law" on subordinate policies, and clarify the connection logic and binding mechanism between superior and subordinate policies.*

*<3> In accordance with the experts' suggestions, add a new chapter to explore the potential thematic structure and evolutionary trends of policies by using the Latent Dirichlet Allocation (LDA) model.*

*<4> Explore the definition of rights and obligations of medical institutions and third-party platforms in policy documents, and clarify the boundary of rights and obligations between the two parties.*

*<5> Compare excellent international policies on online sales of prescription drugs, analyze the gaps between China's existing policies and these excellent international policies, and put forward targeted optimization directions.*

*<6> Conduct sensitivity analysis to explore the impact and mechanism of fluctuations in primary variables on the PMC index.*

*<7> Revise the title of the paper to accurately reflect the correlational nature of this study.*

*<8> Standardize the English translation of "online sales of prescription drugs" throughout the full text, adopting academically standardized expressions to ensure the rigor and consistency of terminology.*

*<9> Conduct a comprehensive revision of the full text, identify and correct errors in grammar, word choice, sentence structure and punctuation, so as to ensure the standardization and fluency of the expression.*

*<10> Unify the format for expressing the release date of policies to enhance the rigor of the paper.*

*<11> The relevant formulas of the entropy weight method are supplemented.*

*<12> Supplement the specific meanings of symbols in all formulas of the paper, clarify the references of variables and parameters, so as to facilitate readers' understanding.*

*<13> Supplement missing citations to provide authoritative literature support for key arguments and methodological choices.*

*<14> Commit to providing supplementary materials such as datasets after the paper is accepted.*

**Reviewer 1** | 5 April 2026 | 12:34

#1. Apply Differential Variable Weighting

The current binary scoring system assigns equal importance to all variables. The authors should incorporate weighting methods such as the Analytic Hierarchy Process (AHP) or the Entropy Weight Method to reflect the varying importance of regulatory dimensions, particularly prioritizing critical aspects such as quality supervision and public safety.

*[Authors’ response] We appreciate your valuable suggestions on differential variable weighting. We fully agree with your observation that the current equal-weight scoring system fails to reflect the varying importance of individual variables, and in particular, cannot adequately highlight the central role of variables related to quality supervision and public safety. In response to your insightful comments, we will implement the following key revisions and additions to the manuscript: Following your recommendation, we have further analyzed the first-level variables using the entropy weight method. We objectively calculated the weight of each variable based on the information entropy of the raw data, thereby avoiding subjective bias caused by artificial weighting and further enhancing the scientific rigor and rationality of our research conclusions.* *In direct response to your specific requirements, we have made the following comprehensive changes:*

*<1>The entropy weight method has been added to the research methodology.*

The entropy weight method is an objective weighting approach based on the principle of information entropy. Its core idea is to determine the weight of each indicator by measuring its degree of dispersion, thereby reflecting the amount of information contained in the data. In this study, the entropy weight method is adopted to assign objective weights to first-level variables, so as to avoid biases caused by subjective assignment and improve the objectivity and scientific rigor of the evaluation results. The main steps are as follows:

① construct the evaluation matrix and standardize the data;

② calculate the information entropy;

③ calculate the difference coefficient based on the information entropy;

④ compute the weight of each indicator and conduct corresponding analysis.

(See lines 124–134.)

*<2 >Add a dedicated paragraph in the Results section (4.4 Indicator Weight Analysis) to present the complete path analysis results, and provide the entropy values and weights of the first-order variables in the newly added Table 4.*

To identify the importance of indicators in the evaluation system for online prescription drug sales policies, this study adopts the entropy weight method to calculate objective weights. Four primary indicators (X2, X3, X4, X10) yield identical values with no distinguishable differences in importance for weighting; therefore, they are excluded, and weight calculations are performed only for the remaining six indicators (See Table 4).

Table 4.

Summary of Weight Calculation Results via the Entropy Method.

| Indicator | X1 | X5 | X6 | X7 | X8 | X9 |
| --- | --- | --- | --- | --- | --- | --- |
| Entropy value | 0.703042 | 0.943769 | 0.657695 | 0.766373 | 0.894784 | 0.948523 |
| Coefficient of Variation | 0.296957 | 0.056231 | 0.342305 | 0.233627 | 0.105216 | 0.051477 |
| Weight | 0.273488 | 0.051787 | 0.315252 | 0.215163 | 0.096901 | 0.047409 |

The results show that indicator X6 has the highest weight, indicating that current policies governing online prescription drug sales prioritize macro-level guidance and regulation. By clarifying industry development boundaries and standardizing the conduct of relevant stakeholders, these policies effectively define the core direction of supervision. Indicator X9 registers the lowest weight, indicating that China has yet to establish a comprehensive full-process closed-loop regulatory system for online prescription drug sales. The linkage between regulatory segments remains inadequate, and cross-departmental collaborative governance mechanisms operate inefficiently. These findings verify that current policies prioritize macro guidance while adopting refined regulation as a supplementary measure.

Future policies should be jointly formulated by the National Medical Products Administration, Ministry of Industry and Information Technology, State Post Bureau, and other relevant authorities. Regulators shall clarify full-process regulatory responsibilities for online prescription drug sales, strengthen cross-agency coordination mechanisms, and improve the comprehensive closed-loop supervision system across the entire industry chain. (See lines 320-352)

#2. Perform a Policy Conflict and Legal Hierarchy Analysis

Given that most policies are departmental regulations with relatively low legislative priority, the authors should analyze potential inconsistencies or conflicts between these documents and higher-level legislation, particularly the Medicinal Product Administration Law. This would provide deeper legal and institutional insights.

*[Authors’ response] We sincerely appreciate your valuable and detailed comments on the policy analysis section of this paper. We have fully incorporated your suggestions by comparing potential conflicts between higher-level laws (the Pharmaceutical Administration Law) and lower-level regulatory documents, and conducting an in-depth analysis of the relevant legal instruments. In direct response to your specific requirements, we have made the following comprehensive changes:*

*<1>In the Horizontal Analysis section (Section 4.2), an in-depth analysis of Dimension X4 is conducted, focusing on exploring the dominant role of superior legal documents in guiding supporting administrative regulations, departmental rules, and other related policy documents.*

Legal Document P10 clarifies the legality of online prescription drug sales at the statutory level. Subsequently, supporting administrative regulations and departmental rules have been successively issued, and in strict accordance with the relevant provisions of the Pharmaceutical Administration Law, targeted regulations have been implemented for core links such as the division of responsibilities, supervision standards, and penalty rules in the field of online prescription drug sales. This evolutionary trajectory of policies fully reflects the guiding and dominant position of superior legal documents centered on the Pharmaceutical Administration Law in the supervision of online prescription drug sales. (See lines 224-241)

#3. Integrate Advanced Topic Modelling Techniques (e.g., LDA)

While keyword frequency and network analysis are informative, employing Latent Dirichlet Allocation (LDA) would allow for the identification of latent thematic structures and policy evolution trends, offering a more sophisticated understanding of regulatory focus over time.

*[Authors’ response] We sincerely appreciate your valuable and detailed comments on the topic modeling and policy analysis sections of this article. We have fully incorporated your suggestions by employing the LDA model to extract themes from policy texts concerning online prescription drug sales and to analyze the evolutionary process of relevant policies, thus strengthening the professionalism and rigor of our study. In direct response to your specific requirements, we have made the following comprehensive changes:*

*<1>Several revisions have been made to the abstract based on the LDA analysis results.*

Amid the continuous deepening of reform and exploration, China’s policy system for the online sale of prescription drugs has been gradually improved. While great emphasis has been placed on the efficiency enabled by smart approaches and the steady development of an intelligent regulatory system, considerable room for optimization remains in key areas including the whole-process regulatory system for online prescription drug sales, the collaborative participation of medical institutions, and the standardized governance of third-party platforms. (See lines 31-37)

*<2>The Latent Dirichlet Allocation (LDA) model is introduced in the research methodology section for topic mining and feature extraction from policy texts.*

Latent Dirichlet Allocation (LDA) is an unsupervised generative probabilistic model widely applied to topic modeling of textual data. This model can effectively identify the topic distributions contained in texts and determine the corresponding word distributions for each individual topic. In this study, Python is employed to construct an LDA model based on 18 policies concerning the online sale of prescription drugs, aiming to identify the underlying thematic structure and evolutionary trends of these policies. (See lines 118-123)

*<3>The Latent Dirichlet Allocation (LDA) model analysis is incorporated in Section 4.5 of the results, through which the potential thematic structure and policy evolution trends in the policy texts are effectively identified, further enhancing the scientificity of the research conclusions and the pertinence of the analysis.*

In this paper, the Latent Dirichlet Allocation (LDA) model is employed to conduct topic mining on policies governing the online sale of prescription drugs. According to the perplexity curve, the optimal number of topics extracted from the policy texts is determined to be three. Based on the feature words under each topic, the three themes are summarized as qualification supervision of online pharmaceutical sales, regulation of online prescription drug transactions, and construction of a digital-intelligent regulatory system, as shown in Table 5. As indicated in Table 2, the theme of qualification supervision of online pharmaceutical sales carries the highest weight, followed by the construction of a digital-intelligent regulatory system, while the regulation of online prescription drug transactions has the lowest weight. This suggests that China’s current policies are relatively well-established in terms of access qualification for online pharmaceutical sales, yet remain inadequate in the whole-process regulation of online prescription drug transactions. This finding is highly consistent with the conclusion from Dimension X9 in Section 4.4 that a comprehensive closed-loop regulatory system for the online sale of prescription drugs has not yet been fully established, verifying the scientific validity and reliability of the evaluation results derived from the PMC index model. Meanwhile, the Chinese government has increasingly recognized the advantages of smart regulation and is continuously advancing the development of a smart regulatory system. Future policies should further clarify the regulatory responsibilities of each link and address weaknesses in whole-process governance. In addition, efforts should be accelerated to establish an integrated digital-intelligent regulatory system, so as to form a highly efficient regulatory framework featuring cross-departmental collaboration, full-chain coverage, and smart governance.

Table 5.

Topic - Feature Words of the 18 Policy Texts

| Theme | Feature Words | Weight /% |
| --- | --- | --- |
| Qualification Supervision of Online Pharmaceutical Sales | Internet, Pharmaceuticals, Websites, Operators, Consumers, Filing, Qualification Certificates, Laws | 44.6 |
| Control of Online Prescription Drug Sales | Platforms, Fines, Medical Institutions, Inspections, Prescriptions, Violations, Sellers, Supervision | 27.2 |
| Construction of Smart Regulatory System | Internet, Reform, Fields, Platforms, Construction, Data, Technology, Supervision, Optimization, Industry, Institutions, System | 28.2 |

(See lines 297-319)

#4. Refine and Expand the “Policy Target” (X5) Dimension

The evaluation of policy objects should be further subdivided to distinguish between regulatory obligations, rights, and accountability mechanisms for medical institutions and third-party platforms. This would help clarify governance responsibilities and enhance analytical precision.

*[Authors’ response] We sincerely appreciate your important and detailed comments on the setting of the primary indicators in this paper. Your point that the current research lacks sufficient refinement in the "strategic objectives" (X5) dimension and insufficient precision in policy object evaluation is completely correct. The regulatory obligations, regulatory rights, and accountability mechanisms involving medical institutions and third-party platforms in X5 have been reflected to a certain extent in the subsequent regulatory system section (X9). In direct response to your specific requirements, we have made the following comprehensive changes:*

*<1>To clearly present the boundary of rights and scope of obligations between medical institutions and third-party platforms, and avoid the repetition of indicator meanings, this paper conducts a systematic analysis on the right allocation and obligation performance of both parties based on the assignment results of secondary indicators in Section 4.2 Horizontal Analysis, so as to ensure the scientificity of the analysis process and the accuracy of the analysis results.*

With an average score of 0.69, dimension X5 indicates that current policies attach great importance to regulating relevant entities involved in online prescription drug sales. However, during the weight assignment process, it was found that among the policies explicitly permitting online prescription drug sales, only four policies (P10, P12, P15, and P16) simultaneously involve medical institutions and third-party platforms, and the relevant provisions fail to clarify the boundaries of rights and obligations between the two parties in online prescription drug sales. Therefore, future policies need to focus on refining the division of responsibilities between medical institutions and third-party platforms, regulating the behaviors of these two types of entities in online prescription drug sales, and enhancing the pertinence and enforceability of the policies. (See lines 231-241)

#5. Conduct International Benchmarking Comparisons

The study would benefit from applying the PMC framework to selected international regulatory systems (e.g., U.S. or EU online pharmacy regulations). Such benchmarking would contextualize China’s policy performance and identify global best practices.

*[Authors’ response] We sincerely appreciate your valuable suggestions regarding the lack of international comparison in this paper. We have fully incorporated your comments by selecting relevant policies from the United States, where online prescription drug sales have a relatively long history, and conducting a comparative analysis with China’s policies using the PMC model developed in this study, thereby enhancing the scientific rigor and international perspective of our research. In direct response to your specific requirements, we have made the following comprehensive changes:*

*<1>In Section 4.3 Vertical Analysis, the PMC framework will be applied to the U.S. Federal Food, Drug, and Cosmetic Act (FDA) to calculate the values of primary variables and the PMC index, which will then be compared with the corresponding values of China's relevant policies.*

In addition, based on the PMC policy evaluation index system, this paper calculates the primary variable values and PMC index of the U.S. Federal Food, Drug, and Cosmetic Act, excluding four single-criterion evaluation primary variables: X2, X3, X4, and X10. The values of the remaining six core primary variables are as follows: X1 is 0.67, and X5, X6, X7, X8, and X9 each have a value of 1. Compared with Policy P11, which is equivalent in legislative hierarchy in China, the US policy lags behind only in two primary variables: X5 and X9. Subsequent policy optimization should focus on addressing regulatory loopholes in the supervision domain and further strengthening standardized constraints on medical institutions and social organizations as participating entities. (See lines 289-296)

#6. Develop a Predictive Policy Roadmap through Sensitivity Analysis

By simulating a “perfect” policy scenario and analyzing which variables most significantly influence the PMC Index, the authors could provide actionable guidance on priority areas for future regulatory reform.

*[Authors’ response] We sincerely appreciate your valuable comments on the policy optimization path section of this paper. We have fully adopted your suggestions by using sensitivity analysis to simulate an "ideal-type" policy scenario and identifying the key variables that have the most significant impact on the PMC index, so as to provide a scientific basis and directional guidance for the subsequent optimization of online prescription drug sales policies. In direct response to your specific requirements, we have made the following comprehensive changes:*

*<1>In the Discussion section, the focus is on analyzing the sensitivity and potential impact of each primary variable on the "perfect" policy scenario, thoroughly examining the impact of fluctuations in different primary variables on the implementation effect of the "perfect" policy, and providing a solid theoretical and empirical basis for policy optimization.*

In addition, the sensitivity analysis conducted on all primary variables reveals that X3 and X4 exert a relatively significant impact on the overall effectiveness of the policy. Notably, when the value of X3 is increased, the PMC index of the optimal policy can reach a maximum of 8.21, which is only 0.29 lower than that of an ideal policy (8.50). When the value of X4 is elevated, the PMC index of the optimal policy peaks at 8.16, representing a gap of 0.34 from the ideal policy. This demonstrates that optimizing the values of X3 and X4 is highly meaningful for policy improvement. However, owing to the fixed nature of policy legal effect and hierarchy, the value of X4 is inherently rigid. Therefore, future policy efforts should be oriented toward the optimization of variable X3, promote joint policy formulation across multiple departments, improve cross‑departmental collaborative governance mechanisms, and enhance the systematicity and coordination of the policy system. (See lines 406-415)

**Reviewer 2** | 6 April 2026 | 12:34

#1. In this paper, the authors present a quantitative assessment of China’s policies on the network sales of prescription drugs based on the PMC index model. The authors should clarify why specific thresholds were used and discuss how these choices may affect the results. It would also be helpful to indicate whether such thresholds could be learned from the data rather than being fixed a priori.

*[Authors’ response] We sincerely appreciate the important and detailed comments you provided on this paper. Regarding the basis for setting the thresholds and their potential impacts on the research results, the specific explanations are as follows: The thresholds adopted in this study are mainly determined based on relevant literature and sensitivity tests. In the specific quantitative analysis of policies, the selection of thresholds directly affects the classification and judgment of policies. To ensure the robustness of the research conclusions, we have conducted multiple sets of sensitivity tests on key thresholds, and performed simulation analysis by adjusting the threshold intervals. The results show that the PMC index values remain consistent within a reasonable range of threshold fluctuations, which proves that the research results of this study have strong reliability and anti-interference ability.*

#2. In addition, the authors should explain why the network structure is not used to weight the PMC index. Since the study employs co-occurrence networks, it could also be useful to discuss related work on this topic, such as 'using complex networks to quantify consistency in the use of words' JSTAT 2012 and 'language clustering with word co-occurrence networks based on parallel texts', Chinese Science Bulletin 2013.

*[Authors’ response] We sincerely appreciate the important and detailed comments you provided on this paper. Regarding the reasons for not using the network structure to weight the PMC index, the specific explanations are as follows: Although the network structure is feasible for weighting the PMC index, this weighting method is prone to being affected by the subjectivity of the network structure, which may lead to deviations. In contrast, the entropy weight method can effectively avoid such subjective interference and ensure the objectivity and rationality of weight allocation. Therefore, in accordance with the comments of Reviewer 1, this study adopts the entropy weight method to explore the weight of primary variables and their importance in the online prescription drug sales policy index system.*

Jelodar H, Wang Y, Yuan C, Feng X, Jiang X, Li Y, et al. Latent Dirichlet allocation (LDA) and topic modeling: models, applications, a survey.Multimedia tools and applications.(2019).78(11), 15169-15211. doi: 10.48550/arXiv.1711.04305

Feng LY ,You QY ,Guan SS .An empirical study on key drivers of blind box online purchase experience based on online reviews: integrating the Kano model and entropy weight method.[J].Scientific reports.(2026).16(1):9743-9743.doi:10.1038/S41598-026-44771-3.
