# Supplementary material for: Quantitative assessment of china’s policies on the online sale of prescription drugs
Source: PLoS One. 2026 Sep 23;21(9):e0358484. doi: 10.1371/journal.pone.0358484 (PMC13600529; doi:10.1371/journal.pone.0358484)
Supplement: Supplementary file 4 [file pone.0358484.s005.docx]

***Summary of major changes:***

*<1>We have conducted a full manuscript review and fixed all defective and invalid in-text citations.*

*<2>We have supplemented and refined the descriptions for robustness tests on threshold selection.*

*<3>We have supplemented the topic coherence scores for the three-topic LDA model to verify the rationality of the optimal number of topics selected in this study.*

*<4>We have corrected misplaced content within the manuscript and reorganized the relevant paragraphs.*

*<5>We have supplemented explanations regarding the rationale for retaining variables X2, X3, X4 and X10 that exhibit no information variance in this study.*

*<6>We have supplemented an explanation that the policy text mining analysis in this study was performed using the original Chinese versions of the policy documents.*

*<7>We have professionally polished the entire manuscript, thoroughly checking and correcting all grammatical and syntactical errors.*

*<8>We have revised our affiliated institution from School of Pharmaceutical Sciences to School of Pharmacy in accordance with the university’s requirements.*

*<9>We have supplemented one new funding source in this revised submission and provided the corresponding updated statement in the cover letter.*

**Reviewer 3**| 29 June 2026 | 16:34

#1. The manuscript contains 'Error! Reference source not found.' placeholders in the text (at lines 49 and 61). The authors must update all cross-references and field codes, perform a global search for 'Error!' and broken citations, and verify that the final PDF renders correctly before resubmission.

*[Authors’ response] We sincerely apologize for the formatting oversights in our manuscript and greatly appreciate the reviewer’s valuable suggestions for revision. In response to the identified formatting issues, we have carefully revised all incorrect cross-references and domain codes throughout the manuscript. Additionally, we have regenerated the PDF file and thoroughly double-checked all citations to ensure complete accuracy and normal display in the revised version.*

#2. Clarify threshold robustness further (Reviewer 2, Point 1).

*[Authors’ response] We sincerely appreciate the reviewer’s suggestion to elaborate on threshold robustness. Following your advice, we conducted multiple rounds of sensitivity tests by integrating our policy sample data with widely accepted threshold criteria adopted in previous policy evaluation studies to validate the robustness of our threshold settings. We have further refined the relevant explanations regarding threshold robustness in the revised manuscript and updated the corresponding content.*

*<1>We have further supplemented and elaborated on the robustness of our threshold settings in Section 3.2.*

Based on the characteristics of the policy samples and established policy evaluation criteria from prior studies^[23,25]^, the stability and rationality of the threshold values were verified through multiple sensitivity tests to develop the PMC index grading standard for policy evaluation in this study. (See lines 171-173)

#3. Report the coherence scores for the 3-topics solution to demonstrate that the LDA model is not merely statistically convenient but semantically meaningful.

*[Authors’ response] We sincerely appreciate the reviewer’s valuable and constructive comments. Following your suggestions, we have supplemented the topic coherence scores for the three-topic LDA model. These metrics confirm that the selected LDA model is not only statistically robust but also delivers practical semantic value in interpreting policy texts. In direct response to your specific requirements, we have made the following comprehensive changes:*

*<1>We have incorporated the coherence scores for the three topics into Table 5 in Section 4.5, which further verifies the rationality of our chosen number of topics.*

The optimal number of topics was determined using two evaluation metrics: the perplexity curve and the topic coherence score. The model achieved an overall average topic coherence score of 0.46. This value falls within a reasonable range due to the limited sample size of policy texts in this study. Combined with the global minimum perplexity value and the semantic interpretability of feature keywords for each topic, three was ultimately selected as the optimal number of topics. (See lines 335-340)

Table 5.

Topic - Feature Words of the 18 Policy Texts

| Theme | Feature Words | Weight /% | Coherence Score |
| --- | --- | --- | --- |
| Qualification Supervision of Online Pharmaceutical Sales | Internet, Pharmaceuticals, Websites, Operators, Consumers, Filing, Qualification Certificates, Laws | 44.6 | 0.41 |
| Control of Online Prescription Drug Sales | Platforms, Fines, Medical Institutions, Inspections, Prescriptions, Violations, Sellers, Supervision | 27.2 | 0.42 |
| Construction of Smart Regulatory System | Internet, Reform, Fields, Platforms, Construction, Data, Technology, Supervision, Optimization, Industry, Institutions, System | 28.2 | 0.54 |

(See lines 358-360)

#4. Section 4.4 (Indicator Weight Analysis) appears after Section 4.5 (LDA Model Analysis). The authors must reorder the manuscript to ensure that all Section 4.x subsections appear consecutively within the Results section.

*[Authors’ response] We sincerely apologize for the sequential formatting errors in our submitted manuscript and appreciate the reviewer’s thoughtful revision suggestions. To address the formatting issues you raised, we have reordered Sections 4.4 (Indicator Weight Analysis) and 4.5 (LDA Model Analysis) in the revised manuscript.*

#5. A brief justification of why X2, X3, X4, and X10 variables are retained in the PMC index, despite having no informational variance for weighting, would strengthen the methodology.

*[Authors’ response] We sincerely appreciate the reviewer’s insightful and constructive comments. We hereby elaborate on why variables X2, X3, X4, and X10 have been retained despite exhibiting insignificant variance during PMC index calculation. These four indicators respectively represent policy constraint intensity, issuing authority tier, legal validity level, and policy disclosure requirements, which serve as core dimensions for assessing the legality and authority of regulatory policies governing the online sales of prescription drugs. Excluding these variables would lead to the absence of key dimensions in the evaluation framework and hinder comprehensive quantitative policy assessment. To guarantee the objectivity and comparability of the horizontal comparative analysis of PMC indices across different policies in Section 4.2, we have retained these four variables and supplemented the corresponding justifications in the methodology section. In direct response to your specific requirements, we have made the following comprehensive changes:*

*<1>In Section 3.1, this study systematically elaborates on the necessity of including each evaluation indicator in the assessment system.*

Aiming to ensure the integrity and rigor of the evaluation system and perform comprehensive quantitative assessments of the included policies, this study established a multidimensional quantitative policy evaluation framework comprising 10 primary variables and 35 secondary variables, as detailed in Table 2. (See lines 149-152)

#6. The language of the text mining is ambiguous, and the authors have not explicitly stated whether the original Chinese policy documents or English-translated versions were analyzed.

*[Authors’ response]We appreciate the reviewer for raising this valuable comment. We acknowledge that the description of data sources used for policy text mining was insufficiently clear in the original manuscript. Herein, we clarify that the original Chinese versions of policy documents were selected as analytical materials for text mining in this study. All relevant keywords and professional terminology were translated with reference to authoritative English legal documents such as the Drug Administration Law of the People's Republic of China to ensure translation accuracy. We have supplemented detailed descriptions in the revised manuscript to clearly elaborate on the above information.*

*<1>We have added relevant clarifications in Section 2.2 (Analysis of Policy Text Mining) to specify that all materials used in this study are the original Chinese versions of policy documents.*

In this study, ROST CM6.0 software was used to conduct in-depth text mining analysis of the original Chinese versions of policies governing the online sale of prescription drugs. (See lines 98-99)

#7. A final, meticulous proofread is absolutely recommended, with particular attention to: (1) grammatical errors and awkward phrasing throughout; (2) inconsistent notation in equations (especially the index j in the Xtj term of Equation 2); and (3) broken cross-references to figures and tables.

*[Authors’ response] We sincerely apologize for the grammatical and formatting oversights in the original manuscript. We are grateful for your meticulous review and the careful identification of these issues. We have conducted a thorough final proofreading of the entire manuscript, revising grammatical errors, inconsistent formula symbols, and invalid cross-references for all figures and tables throughout the text.*

#8. According to PLOS ONE's policy, the "minimal data set" includes the data required to replicate all study findings. The supplementary materials provided are insufficient to fully support the authors' Data Availability Statement that all data are fully available without restriction.

*[Authors’ response] We sincerely appreciate the reviewer’s careful and valuable comments. In response to the issue that the currently submitted supplementary materials fail to support the statement of unrestricted public access to all data in our data availability section and do not meet the PLOS ONE data policy requirements for a minimal dataset that can fully reproduce all research findings, we have made the following revisions: First, we have reorganized the minimal dataset that can completely replicate all the research results in strict accordance with PLOS ONE’s data policy and supplemented these data into the supplementary materials, so that other researchers can repeat our entire empirical process and reproduce all study conclusions based on the submitted dataset. Second, we have revised the data availability statement accordingly. We removed the wording regarding unrestricted public access to all data and accurately described the scope of publicly available data and access permissions based on the newly uploaded minimal dataset, ensuring that the data availability statement is fully consistent with the supplementary materials we submitted.*
