# Supplementary material for: Quantitative assessment of china’s policies on the online sale of prescription drugs
Source: PLoS One. 2026 Sep 23;21(9):e0358484. doi: 10.1371/journal.pone.0358484 (PMC13600529; doi:10.1371/journal.pone.0358484)
Supplement: Supplementary file 5 [file pone.0358484.s006.docx]

**Recommendation 1: Threshold robustness**

The added sentence in Section 3.2 only asserts that thresholds were validated "through multiple sensitivity tests" without showing any of the actual results. The authors should add concrete, verifiable content to Section 3.2, such as:

- The specific threshold values tested (e.g., alternative cut-points for "Excellent," "Good," etc.) and the source studies each was drawn from.
- The outcome of the sensitivity tests, for example, a table or brief narrative showing how many policies would shift classification (and by how much) under each alternative threshold set, demonstrating that the final grading bands are stable.
- A brief explanation of the criterion used to select the final thresholds over the alternatives (e.g., minimal reclassification, best alignment with sample distribution, closest match to prior literature).

**Recommendation 2: Justification for retaining X2, X3, X4, X10**

The rationale given in the response letter (that these four variables represent policy constraint intensity, issuing authority tier, legal validity level, and policy disclosure requirements, and are core to assessing legality/authority) needs to actually be inserted into Section 3.1 of the manuscript it currently only appears in the response letter.
