# Supplementary material for: Quantitative assessment of china’s policies on the online sale of prescription drugs
Source: PLoS One. 2026 Sep 23;21(9):e0358484. doi: 10.1371/journal.pone.0358484 (PMC13600529; doi:10.1371/journal.pone.0358484)
Supplement: Supplementary file 6 [file pone.0358484.s007.docx]

***Summary of major changes:***

*<1>In response to the reviewer’s comments, we have supplemented detailed and verifiable sensitivity test results to strengthen the empirical evidence supporting the stability of the thresholds.*

*<2> In accordance with the reviewer’s suggestion, we have incorporated the rationale for retaining variables X2, X3, X4 and X10 into the manuscript.*

**Reviewer 1**| 8 August 2026 | 16:34

#1: Recommendation 1: Threshold robustness

The added sentence in Section 3.2 only asserts that thresholds were validated "through multiple sensitivity tests" without showing any of the actual results. The authors should add concrete, verifiable content to Section 3.2, such as:

1. The specific threshold values tested (e.g., alternative cut-points for "Excellent," "Good," etc.) and the source studies each was drawn from.

2. The outcome of the sensitivity tests, for example, a table or brief narrative showing how many policies would shift classification (and by how much) under each alternative threshold set, demonstrating that the final grading bands are stable.

3. A brief explanation of the criterion used to select the final thresholds over the alternatives (e.g., minimal reclassification, best alignment with sample distribution, closest match to prior literature).

*[Authors’ response] We sincerely appreciate the reviewer’s insightful and constructive comments. This study developed the grading standard for the PMC index based on the two core cluster centers, which were derived from cluster analysis of PMC index results using SPSS 27.0, and evaluation criteria reported in prior literature. In addition, we have systematically supplemented the sensitivity test results in Section 3.2 to fully verify the rationality and robustness of this grading standard. The detailed revisions are presented below.*

*<1>Add detailed empirical results of sensitivity tests in Section 3.2 to verify the robustness of the grading standard.*

Based on the two core cluster centers (5.81, 6.75), which were derived from cluster analysis of PMC index results using SPSS 27.0, and policy evaluation criteria proposed in prior literature^[23,25]^, this study developed a grading standard for the PMC index: PMC index values from 8.50 to 10.00 correspond to "Perfect", 6.50–8.49 to "Excellent", 5.00–6.49 to "Good", and 0.00–4.99 to "Non-compliant". To test the robustness of this grading standard, two alternative schemes with moderately adjusted cut-points around the baseline critical values were constructed for sensitivity analysis. Scheme A: Perfect [8.30, 10.00], Excellent [6.30, 8.29], Good [4.80, 6.29], Non-compliant [0.00, 4.79]; Scheme B: Perfect [8.70, 10.00], Excellent [6.70, 8.69], Good [5.20, 6.69], Non-compliant [0.00, 5.19]. The results indicate that three policies experience grade shifts under Scheme A, while only one policy undergoes a grade adjustment under Scheme B. No samples fall into the Perfect or Non-compliant intervals. The low proportion of sample reclassification across alternative schemes demonstrates that the defined grading bands exhibit satisfactory robustness. Therefore, the original threshold set is adopted as the grading criterion for policy evaluation in this study. (See lines 174-186)

Recommendation 2: Justification for retaining X2, X3, X4, X10

The rationale given in the response letter (that these four variables represent policy constraint intensity, issuing authority tier, legal validity level, and policy disclosure requirements, and are core to assessing legality/authority) needs to actually be inserted into Section 3.1 of the manuscript it currently only appears in the response letter.

*[Authors’ response] We greatly appreciate the reviewer’s valuable suggestion. We fully agree with this comment and have formally incorporated the rationale for retaining variables X2, X3, X4, and X10 into the revised manuscript.*

*<1>In Section 3.1, this study systematically elaborates on the necessity of including each evaluation indicator in the assessment system.*

Among these indicators, X2, X3, X4, and X10 respectively represent policy constraint intensity, issuing authority tier, legal validity level, and policy disclosure requirements, which serve as core dimensions for assessing the legality and authority of regulatory policies governing the online sales of prescription drugs. (See lines 149-152)
