# Supplementary material for: Quantitative assessment of china’s policies on the online sale of prescription drugs
Source: PLoS One. 2026 Sep 23;21(9):e0358484. doi: 10.1371/journal.pone.0358484 (PMC13600529; doi:10.1371/journal.pone.0358484)
Supplement: Peer review history

# Peer review: Decision Letter 0

**DOI:** 10.1371/journal.pone.0358484.r001

**Authors:** Godwin Banafo Akrong

16 Mar 2026

Dear Dr. Xie,

Thank you for submitting your manuscript to PLOS ONE. After careful consideration, we feel that it has merit but does not fully meet PLOS ONE’s publication criteria as it currently stands. Therefore, we invite you to submit a revised version of the manuscript that addresses the points raised during the review process.

==============================

I encourage you to address all the reviewers’ (1# and #2) comments and make the necessary revisions. I look forward to reviewing your revised manuscript.<o:p></o:p>

Please submit your revised manuscript by Apr 30 2026 11:59PM. If you will need more time than this to complete your revisions, please reply to this message or contact the journal office at plosone@plos.org. When you're ready to submit your revision, log on to https://www.editorialmanager.com/pone/ and select the 'Submissions Needing Revision' folder to locate your manuscript file.

- A letter that responds to each point raised by the academic editor and reviewer(s). You should upload this letter as a separate file labeled 'Response to Reviewers'.
- A marked-up copy of your manuscript that highlights changes made to the original version. You should upload this as a separate file labeled 'Revised Manuscript with Track Changes'.
- An unmarked version of your revised paper without tracked changes. You should upload this as a separate file labeled 'Manuscript'.

If applicable, we recommend that you deposit your laboratory protocols in protocols.io to enhance the reproducibility of your results. Protocols.io assigns your protocol its own identifier (DOI) so that it can be cited independently in the future. For instructions see: https://journals.plos.org/plosone/s/submission-guidelines#loc-laboratory-protocols. Additionally, PLOS ONE offers an option for publishing peer-reviewed Lab Protocol articles, which describe protocols hosted on protocols.io. Read more information on sharing protocols at https://plos.org/protocols?utm_medium=editorial-email&utm_source=authorletters&utm_campaign=protocols.

We look forward to receiving your revised manuscript.

Kind regards,

Godwin Banafo Akrong, Ph.D.

Academic Editor

PLOS One

**Journal Requirements:**

When submitting your revision, we need you to address these additional requirements.

1. Please ensure that your manuscript meets PLOS ONE's style requirements, including those for file naming. The PLOS ONE style templates can be found at

https://journals.plos.org/plosone/s/file?id=wjVg/PLOSOne_formatting_sample_main_body.pdf and

https://journals.plos.org/plosone/s/file?id=ba62/PLOSOne_formatting_sample_title_authors_affiliations.pdf

2. Thank you for stating the following financial disclosure:

“This research was supported by the 2021 Collaborative Innovation Project for Major Scientific Research Projects in Anhui Provincial Universities (Natural Sciences) (grant no. GXXT-2021-068) and the Regulatory Science Research on Online Sales of Pharmaceuticals and Medical Devices (Administrative Research Project) (grant no. AHYJ-KJ-202301).”

Please state what role the funders took in the study.  If the funders had no role, please state: "The funders had no role in study design, data collection and analysis, decision to publish, or preparation of the manuscript."

If this statement is not correct you must amend it as needed.

Please include this amended Role of Funder statement in your cover letter; we will change the online submission form on your behalf.

3. Please upload a new copy of Figures 1, 2a, 2b, 2c and 2d as the detail is not clear. Please follow the link for more information:  https://journals.plos.org/plosone/s/figures

4. Please include captions for your Supporting Information files at the end of your manuscript, and update any in-text citations to match accordingly. Please see our Supporting Information guidelines for more information: http://journals.plos.org/plosone/s/supporting-information.

5. If the reviewer comments include a recommendation to cite specific previously published works, please review and evaluate these publications to determine whether they are relevant and should be cited. There is no requirement to cite these works unless the editor has indicated otherwise.

6. Please review your reference list to ensure that it is complete and correct. If you have cited papers that have been retracted, please include the rationale for doing so in the manuscript text, or remove these references and replace them with relevant current references. Any changes to the reference list should be mentioned in the rebuttal letter that accompanies your revised manuscript. If you need to cite a retracted article, indicate the article’s retracted status in the References list and also include a citation and full reference for the retraction notice.

[Note: HTML markup is below. Please do not edit.]

Reviewers' comments:

Reviewer's Responses to Questions

**Comments to the Author**

1. Is the manuscript technically sound, and do the data support the conclusions?

Reviewer #1: Yes

Reviewer #2: Partly

**********

2. Has the statistical analysis been performed appropriately and rigorously?

Reviewer #1: Yes

Reviewer #2: I Don't Know

**********

3. Have the authors made all data underlying the findings in their manuscript fully available?

The PLOS Data policy

Reviewer #1: No

Reviewer #2: Yes

**********

4. Is the manuscript presented in an intelligible fashion and written in standard English?

Reviewer #1: Yes

Reviewer #2: Yes

**********

Reviewer #1: QUANTITATIVE ASSESSMENT OF CHINA’S POLICIES ON THE NETWORK SALES OF PRESCRIPTION DRUGS BASED ON THE PMC INDEX MODEL: REVIEWER COMMENTS

General Comments

This study addresses a critical and timely issue by quantitatively evaluating China's regulatory policies on the online sales of prescription drugs through a structured PMC Index Model combined with text mining techniques. In light of the rapid expansion of digital healthcare platforms and the associated public safety risks, the authors' systematic assessment of policy consistency and regulatory comprehensiveness is highly valuable. The integration of policy text analysis with quantitative modeling offers a clear and replicable framework for policy evaluation, and the findings provide meaningful insights into both the progress and existing gaps within China's evolving regulatory system. Overall, the research makes a significant contribution to the literature on digital health governance and public policy evaluation.

Recommendations for Improvement

To further enhance the rigor, analytical depth, and policy relevance of the study, the following expert recommendations are proposed:

1. Apply Differential Variable Weighting

The current binary scoring system assigns equal importance to all variables. The authors should incorporate weighting methods such as the Analytic Hierarchy Process (AHP) or the Entropy Weight Method to reflect the varying importance of regulatory dimensions, particularly prioritizing critical aspects such as quality supervision and public safety.

2. Perform a Policy Conflict and Legal Hierarchy Analysis

Given that most policies are departmental regulations with relatively low legislative priority, the authors should analyze potential inconsistencies or conflicts between these documents and higher-level legislation, particularly the Medicinal Product Administration Law. This would provide deeper legal and institutional insights.

3. Integrate Advanced Topic Modelling Techniques (e.g., LDA)

While keyword frequency and network analysis are informative, employing Latent Dirichlet Allocation (LDA) would allow for the identification of latent thematic structures and policy evolution trends, offering a more sophisticated understanding of regulatory focus over time.

4. Refine and Expand the “Policy Target” (X5) Dimension

The evaluation of policy objects should be further subdivided to distinguish between regulatory obligations, rights, and accountability mechanisms for medical institutions and third-party platforms. This would help clarify governance responsibilities and enhance analytical precision.

5. Conduct International Benchmarking Comparisons

The study would benefit from applying the PMC framework to selected international regulatory systems (e.g., U.S. or EU online pharmacy regulations). Such benchmarking would contextualize China’s policy performance and identify global best practices.

6. Develop a Predictive Policy Roadmap through Sensitivity Analysis

By simulating a “perfect” policy scenario and analyzing which variables most significantly influence the PMC Index, the authors could provide actionable guidance on priority areas for future regulatory reform.

Concluding Statement

This is a promising and well-structured study that offers valuable quantitative insights into the regulation of online prescription drug sales in China. With the incorporation of the recommended methodological enhancements and deeper analytical components, the manuscript has strong potential to make a substantial contribution to policy evaluation research. I recommend that the study be accepted for publication following satisfactory revision addressing the above points.

Reviewer #2: In this paper, the authors present a quantitative assessment of China’s policies on the network sales of prescription drugs based on the PMC index model. The authors should clarify why specific thresholds were used and discuss how these choices may affect the results. It would also be helpful to indicate whether such thresholds could be learned from the data rather than being fixed a priori. In addition, the authors should explain why the network structure is not used to weight the PMC index. Since the study employs co-occurrence networks, it could also be useful to discuss related work on this topic, such as 'using complex networks to quantify consistency in the use of words' JSTAT 2012 and 'language clustering with word co-occurrence networks based on parallel texts', Chinese Science Bulletin 2013.

**********

what does this mean?). If published, this will include your full peer review and any attached files.

If you choose “no”, your identity will remain anonymous but your review may still be made public.

**Do you want your identity to be public for this peer review?**  For information about this choice, including consent withdrawal, please see our Privacy Policy

Reviewer #1: No

Reviewer #2: No

**********

[NOTE: If reviewer comments were submitted as an attachment file, they will be attached to this email and accessible via the submission site. Please log into your account, locate the manuscript record, and check for the action link "View Attachments". If this link does not appear, there are no attachment files.]

To ensure your figures meet our technical requirements, please review our figure guidelines: https://journals.plos.org/plosone/s/figures

You may also use PLOS’s free figure tool, NAAS, to help you prepare publication quality figures: https://journals.plos.org/plosone/s/figures#loc-tools-for-figure-preparation.

NAAS will assess whether your figures meet our technical requirements by comparing each figure against our figure specifications.

Attachment Submitted filename: REVIEWER COMMENTS.docx

# Author comment: Author response to Decision Letter 1

**DOI:** 10.1371/journal.pone.0358484.r002

4 May 2026

We sincerely thank the reviewers for their insightful and constructive feedback, which has greatly strengthened the quality and rigor of our manuscript. We have thoroughly addressed all concerns, with detailed point-by-point responses and corresponding manuscript revisions provided below. All figures have been standardized per NAAS guidelines and submitted accordingly.

Summary of major changes:

<1> In accordance with the experts' suggestions, add a new chapter to explore the weight of primary indicators by using the entropy weight method, and analyze their paths and implications for policy optimization.

<2> Analyze the binding force of superior legislation such as the "Drug Administration Law" on subordinate policies, and clarify the connection logic and binding mechanism between superior and subordinate policies.

<3> In accordance with the experts' suggestions, add a new chapter to explore the potential thematic structure and evolutionary trends of policies by using the Latent Dirichlet Allocation (LDA) model.

<4> Explore the definition of rights and obligations of medical institutions and third-party platforms in policy documents, and clarify the boundary of rights and obligations between the two parties.

<5> Compare excellent international policies on online sales of prescription drugs, analyze the gaps between China's existing policies and these excellent international policies, and put forward targeted optimization directions.

<6> Conduct sensitivity analysis to explore the impact and mechanism of fluctuations in primary variables on the PMC index.

<7> Revise the title of the paper to accurately reflect the correlational nature of this study.

<8> Standardize the English translation of "online sales of prescription drugs" throughout the full text, adopting academically standardized expressions to ensure the rigor and consistency of terminology.

<9> Conduct a comprehensive revision of the full text, identify and correct errors in grammar, word choice, sentence structure and punctuation, so as to ensure the standardization and fluency of the expression.

<10> Unify the format for expressing the release date of policies to enhance the rigor of the paper.

<11> The relevant formulas of the entropy weight method are supplemented.

<12> Supplement the specific meanings of symbols in all formulas of the paper, clarify the references of variables and parameters, so as to facilitate readers' understanding.

<13> Supplement missing citations to provide authoritative literature support for key arguments and methodological choices.

<14> Commit to providing supplementary materials such as datasets after the paper is accepted.

Summary of major changes:

Reviewer 1

#1. Apply Differential Variable Weighting

The current binary scoring system assigns equal importance to all variables. The authors should incorporate weighting methods such as the Analytic Hierarchy Process (AHP) or the Entropy Weight Method to reflect the varying importance of regulatory dimensions, particularly prioritizing critical aspects such as quality supervision and public safety.

[Authors’ response] We appreciate your valuable suggestions on differential variable weighting. We fully agree with your observation that the current equal-weight scoring system fails to reflect the varying importance of individual variables, and in particular, cannot adequately highlight the central role of variables related to quality supervision and public safety. In response to your insightful comments, we will implement the following key revisions and additions to the manuscript: Following your recommendation, we have further analyzed the first-level variables using the entropy weight method. We objectively calculated the weight of each variable based on the information entropy of the raw data, thereby avoiding subjective bias caused by artificial weighting and further enhancing the scientific rigor and rationality of our research conclusions. In direct response to your specific requirements, we have made the following comprehensive changes:

<1>The entropy weight method has been added to the research methodology.

The entropy weight method is an objective weighting approach based on the principle of information entropy. Its core idea is to determine the weight of each indicator by measuring its degree of dispersion, thereby reflecting the amount of information contained in the data. In this study, the entropy weight method is adopted to assign objective weights to first-level variables, so as to avoid biases caused by subjective assignment and improve the objectivity and scientific rigor of the evaluation results. The main steps are as follows:

① construct the evaluation matrix and standardize the data;

② calculate the information entropy;

③ calculate the difference coefficient based on the information entropy;

④ compute the weight of each indicator and conduct corresponding analysis.

(See lines 124–134.)

<2 >Add a dedicated paragraph in the Results section (4.4 Indicator Weight Analysis) to present the complete path analysis results, and provide the entropy values and weights of the first-order variables in the newly added Table 4.

To identify the importance of indicators in the evaluation system for online prescription drug sales policies, this study adopts the entropy weight method to calculate objective weights. Four primary indicators (X2, X3, X4, X10) yield identical values with no distinguishable differences in importance for weighting; therefore, they are excluded, and weight calculations are performed only for the remaining six indicators (See Table 4).

Table 4.

Summary of Weight Calculation Results via the Entropy Method.

Indicator X1 X5 X6 X7 X8 X9

Entropy value 0.703042 0.943769 0.657695 0.766373 0.894784 0.948523

Coefficient of Variation 0.296957 0.056231 0.342305 0.233627 0.105216 0.051477

Weight 0.273488 0.051787 0.315252 0.215163 0.096901 0.047409

The results show that indicator X6 has the highest weight, indicating that current policies governing online prescription drug sales prioritize macro-level guidance and regulation. By clarifying industry development boundaries and standardizing the conduct of relevant stakeholders, these policies effectively define the core direction of supervision. Indicator X9 registers the lowest weight, indicating that China has yet to establish a comprehensive full-process closed-loop regulatory system for online prescription drug sales. The linkage between regulatory segments remains inadequate, and cross-departmental collaborative governance mechanisms operate inefficiently. These findings verify that current policies prioritize macro guidance while adopting refined regulation as a supplementary measure.

Future policies should be jointly formulated by the National Medical Products Administration, Ministry of Industry and Information Technology, State Post Bureau, and other relevant authorities. Regulators shall clarify full-process regulatory responsibilities for online prescription drug sales, strengthen cross-agency coordination mechanisms, and improve the comprehensive closed-loop supervision system across the entire industry chain. (See lines 320-352)

#2. Perform a Policy Conflict and Legal Hierarchy Analysis

Given that most policies are departmental regulations with relatively low legislative priority, the authors should analyze potential inconsistencies or conflicts between these documents and higher-level legislation, particularly the Medicinal Product Administration Law. This would provide deeper legal and institutional insights.

[Authors’ response] We sincerely appreciate your valuable and detailed comments on the policy analysis section of this paper. We have fully incorporated your suggestions by comparing potential conflicts between higher-level laws (the Pharmaceutical Administration Law) and lower-level regulatory documents, and conducting an in-depth analysis of the relevant legal instruments. In direct response to your specific requirements, we have made the following comprehensive changes:

<1>In the Horizontal Analysis section (Section 4.2), an in-depth analysis of Dimension X4 is conducted, focusing on exploring the dominant role of superior legal documents in guiding supporting administrative regulations, departmental rules, and other related policy documents.

Legal Document P10 clarifies the legality of online prescription drug sales at the statutory level. Subsequently, supporting administrative regulations and departmental rules have been successively issued, and in strict accordance with the relevant provisions of the Pharmaceutical Administration Law, targeted regulations have been implemented for core links such as the division of responsibilities, supervision standards, and penalty rules in the field of online prescription drug sales. This evolutionary trajectory of policies fully reflects the guiding and dominant position of superior legal documents centered on the Pharmaceutical Administration Law in the supervision of online prescription drug sales. (See lines 224-241)

#3. Integrate Advanced Topic Modelling Techniques (e.g., LDA)

While keyword frequency and network analysis are informative, employing Latent Dirichlet Allocation (LDA) would allow for the identification of latent thematic structures and policy evolution trends, offering a more sophisticated understanding of regulatory focus over time.

[Authors’ response] We sincerely appreciate your valuable and detailed comments on the topic modeling and policy analysis sections of this article. We have fully incorporated your suggestions by employing the LDA model to extract themes from policy texts concerning online prescription drug sales and to analyze the evolutionary process of relevant policies, thus strengthening the professionalism and rigor of our study. In direct response to your specific requirements, we have made the following comprehensive changes:

<1>Several revisions have been made to the abstract based on the LDA analysis results.

Amid the continuous deepening of reform and exploration, China’s policy system for the online sale of prescription drugs has been gradually improved. While great emphasis has been placed on the efficiency enabled by smart approaches and the steady development of an intelligent regulatory system, considerable room for optimization remains in key areas including the whole-process regulatory system for online prescription drug sales, the collaborative participation of medical institutions, and the standardized governance of third-party platforms. (See lines 31-37)

<2>The Latent Dirichlet Allocation (LDA) model is introduced in the research methodology section for topic mining and feature extraction from policy texts.

Latent Dirichlet Allocation (LDA) is an unsupervised generative probabilistic model widely applied to topic modeling of textual data. This model can effectively identify the topic distributions contained in texts and determine the corresponding word distributions for each individual topic. In this study, Python is employed to construct an LDA model based on 18 policies concerning the online sale of prescription drugs, aiming to identify the underlying thematic structure and evolutionary trends of these policies. (See lines 118-123)

<3>The Latent Dirichlet Allocation (LDA) model analysis is incorporated in Section 4.5 of the results, through which the potential thematic structure and policy evolution trends in the policy texts are effectively identified, further enhancing the scientificity of the research conclusions and the pertinence of the analysis.

In this paper, the Latent Dirichlet Allocation (LDA) model is employed to conduct topic mining on policies governing the online sale of prescription drugs. According to the perplexity curve, the optimal number of topics extracted from the policy texts is determined to be three. Based on the feature words under each topic, the three themes are summarized as qualification supervision of online pharmaceutical sales, regulation of online prescription drug transactions, and construction of a digital-intelligent regulatory system, as shown in Table 5. As indicated in Table 2, the theme of qualification supervision of online pharmaceutical sales carries the highest weight, followed by the construction of a digital-intelligent regulatory system, while the regulation of online prescription drug transactions has the lowest weight. This suggests that China’s current policies are relatively well-established in terms of access qualification for online pharmaceutical sales, yet remain inadequate in the whole-process regulation of online prescription drug transactions. This finding is highly consistent with the conclusion from Dimension X9 in Section 4.4 that a comprehensive closed-loop regulatory system for the online sale of prescription drugs has not yet been fully established, verifying the scientific validity and reliability of the evaluation results derived from the PMC index model. Meanwhile, the Chinese government has increasingly recognized the advantages of smart regulation and is continuously advancing the development of a smart regulatory system. Future policies should further clarify the regulatory responsibilities of each link and address weaknesses in whole-process governance. In addition, efforts should be accelerated to establish an integrated digital-intelligent regulatory system, so as to form a highly efficient regulatory framework featuring cross-departmental collaboration, full-chain coverage, and smart governance.

Table 5.

Topic - Feature Words of the 18 Policy Texts

Theme Feature Words Weight /%

Qualification Supervision of Online Pharmaceutical Sales Internet, Pharmaceuticals, Websites, Operators, Consumers, Filing, Qualification Certificates, Laws 44.6

Control of Online Prescription Drug Sales Platforms, Fines, Medical Institutions, Inspections, Prescriptions, Violations, Sellers, Supervision 27.2

Construction of Smart Regulatory System Internet, Reform, Fields, Platforms, Construction, Data, Technology, Supervision, Optimization, Industry, Institutions, System 28.2

(See lines 297-319)

#4. Refine and Expand the “Policy Target” (X5) Dimension

The evaluation of policy objects should be further subdivided to distinguish between regulatory obligations, rights, and accountability mechanisms for medical institutions and third-party platforms. This would help clarify governance responsibilities and enhance analytical precision.

[Authors’ response] We sincerely appreciate your important and detailed comments on the setting of the primary indicators in this paper. Your point that the current research lacks sufficient refinement in the "strategic objectives" (X5) dimension and insufficient precision in policy object evaluation is completely correct. The regulatory obligations, regulatory rights, and accountability mechanisms involving medical institutions and third-party platforms in X5 have been reflected to a certain extent in the subsequent regulatory system section (X9). In direct response to your specific requirements, we have made the following comprehensive changes:

<1>To clearly present the boundary of rights and scope of obligations between medical institutions and third-party platforms, and avoid the repetition of indicator meanings, this paper conducts a systematic analysis on the right allocation and obligation performance of both parties based on the assignment results of secondary indicators in Section 4.2 Horizontal Analysis, so as to ensure the scientificity of the analysis process and the accuracy of the analysis results.

With an average score of 0.69, dimension X5 indicates that current policies attach great importance to regulating relevant entities involved in online prescription drug sales. However, during the weight assignment process, it was found that among the policies explicitly permitting online prescription drug sales, only four policies (P10, P12, P15, and P16) simultaneously involve medical institutions and third-party platforms, and the relevant provisions fail to clarify the boundaries of rights and obligations between the two parties in online prescription drug sales. Therefore, future policies need to focus on refining the division of responsibilities between medical institutions and third-party platforms, regulating the behaviors of these two types of entities in online prescription drug sales, and enhancing the pertinence and enforceability of the policies. (See lines 231-241)

#5. Conduct International Benchmarking Comparisons

The study would benefit from applying the PMC framework to selected international regulatory systems (e.g., U.S. or EU online pharmacy regulations). Such benchmarking would contextualize China’s policy performance and identify global best practices.

[Authors’ response] We sincerely appreciate your valuable suggestions regarding the lack of international comparison in this paper. We have fully incorporated your comments by selecting relevant policies from the United States, where online prescription drug sales have a relatively long history, and conducting a comparative analysis with China’s policies using the PMC model developed in this study, thereby enhancing the scientific rigor and international perspective of our research. In direct response to your specific requirements, we have made the following comprehensive changes:

<1>In Section 4.3 Vertical Analysis, the PMC framework will be applied to the U.S. Federal Food, Drug, and Cosmetic Act (FDA) to calculate the values of primary variables and the PMC index, which will then be compared with the corresponding values of China's relevant policies.

In addition, based on the PMC policy evaluation index system, this paper calculates the primary variable values and PMC index of the U.S. Federal Food, Drug, and Cosmetic Act, excluding four single-criterion evaluation primary variables: X2, X3, X4, and X10. The values of the remaining six core primary variables are as follows: X1 is 0.67, and X5, X6, X7, X8, and X9 each have a value of 1. Compared with Policy P11, which is equivalent in legislative hierarchy in China, the US policy lags behind only in two primary variables: X5 and X9. Subsequent policy optimization should focus on addressing regulatory loopholes in the supervision domain and further strengthening standardized constraints on medical institutions and social organizations as participating entities. (See lines 289-296)

#6. Develop a Predictive Policy Roadmap through Sensitivity Analysis

By simulating a “perfect” policy scenario and analyzing which variables most significantly influence the PMC Index, the authors could provide actionable guidance on priority areas for future regulatory reform.

[Authors’ response] We sincerely appreciate your valuable comments on the policy optimization path section of this paper. We have fully adopted your suggestions by using sensitivity analysis to simulate an "ideal-type" policy scenario and identifying the key variables that have the most significant impact on the PMC index, so as to provide a scientific basis and directional guidance for the subsequent optimization of online prescription drug sales policies. In direct response to your specific requirements, we have made the following comprehensive changes:

<1>In the Discussion section, the focus is on analyzing the sensitivity and potential impact of each primary variable on the "perfect" policy scenario, thoroughly examining the impact of fluctuations in different primary variables on the implementation effect of the "perfect" policy, and providing a solid theoretical and empirical basis for policy optimization.

In addition, the sensitivity analysis conducted on all primary variables reveals that X3 and X4 exert a relatively significant impact on the overall effectiveness of the policy. Notably, when the value of X3 is increased, the PMC index of the optimal policy can reach a maximum of 8.21, which is only 0.29 lower than that of an ideal policy (8.50). When the value of X4 is elevated, the PMC index of the optimal policy peaks at 8.16, representing a gap of 0.34 from the ideal policy. This demonstrates that optimizing the values of X3 and X4 is highly meaningful for policy improvement. However, owing to the fixed nature of policy legal effect and hierarchy, the value of X4 is inherently rigid. Therefore, future policy efforts should be oriented toward the optimization of variable X3, promote joint policy formulation across multiple departments, improve cross departmental collaborative governance mechanisms, and enhance the systematicity and coordination of the policy system. (See lines 406-415)

Reviewer 2

#1. In this paper, the authors present a quantitative assessment of China’s policies on the network sales of prescription drugs based on the PMC index model. The authors should clarify why specific thresholds were used and discuss how these choices may affect the results. It would also be helpful to indicate whether such thresholds could be learned from the data rather than being fixed a priori.

[Authors’ response] We sincerely appreciate the important and detailed comments you provided on this paper. Regarding the basis for setting the thresholds and their potential impacts on the research results, the specific explanations are as follows: The thresholds adopted in this study are mainly determined based on relevant literature and sensitivity tests. In the specific quantitative analysis of policies, the selection of thresholds directly affects the classification and judgment of policies. To ensure the robustness of the research conclusions, we have conducted multiple sets of sensitivity tests on key thresholds, and performed simulation analysis by adjusting the threshold intervals. The results show that the PMC index values remain consistent within a reasonable range of threshold fluctuations, which proves that the research results of this study have strong reliability and anti-interference ability.

#2. In addition, the authors should explain why the network structure is not used to weight the PMC index. Since the study employs co-occurrence networks, it could also be useful to discuss related work on this topic, such as 'using complex networks to quantify consistency in the use of words' JSTAT 2012 and 'language clustering with word co-occurrence networks based on parallel texts', Chinese Science Bulletin 2013.

[Authors’ response] We sincerely appreciate the important and detailed comments you provided on this paper. Regarding the reasons for not using the network structure to weight the PMC index, the specific explanations are as follows: Although the network structure is feasible for weighting the PMC index, this weighting method is prone to being affected by the subjectivity of the network structure, which may lead to deviations. In contrast, the entropy weight method can effectively avoid such subjective interference and ensure the objectivity and rationality of weight allocation. Therefore, in accordance with the comments of Reviewer 1, this study adopts the entropy weight method to explore the weight of primary variables and their importance in the online prescription drug sales policy index system.

Jelodar H, Wang Y, Yuan C, Feng X, Jiang X, Li Y, et al. Latent Dirichlet allocation (LDA) and topic modeling: models, applications, a survey.Multimedia tools and applications.(2019).78(11), 15169-15211. doi: 10.48550/arXiv.1711.04305

Feng LY ,You QY ,Guan SS .An empirical study on key drivers of blind box online purchase experience based on online reviews: integrating the Kano model and entropy weight method.[J].Scientific reports.(2026).16(1):9743-9743.doi:10.1038/S41598-026-44771-3.

Attachment Submitted filename: renamed_07e2c.docx

# Peer review: Decision Letter 1

**DOI:** 10.1371/journal.pone.0358484.r003

**Authors:** Godwin Banafo Akrong

25 Jun 2026

Dear Dr. Xue-Feng,

Thank you for submitting your manuscript to PLOS ONE. After careful consideration, we feel that it has merit but does not fully meet PLOS ONE’s publication criteria as it currently stands. Therefore, we invite you to submit a revised version of the manuscript that addresses the points raised during the review process.

==============================

Please submit your revised manuscript by Aug 09 2026 11:59PM. If you will need more time than this to complete your revisions, please reply to this message or contact the journal office at plosone@plos.org. When you're ready to submit your revision, log on to https://www.editorialmanager.com/pone/ and select the 'Submissions Needing Revision' folder to locate your manuscript file.

- A letter that responds to each point raised by the academic editor and reviewer(s). You should upload this letter as a separate file labeled 'Response to Reviewers'.
- A marked-up copy of your manuscript that highlights changes made to the original version. You should upload this as a separate file labeled 'Revised Manuscript with Track Changes'.
- An unmarked version of your revised paper without tracked changes. You should upload this as a separate file labeled 'Manuscript'.

If you would like to make changes to your financial disclosure, please include your updated statement in your cover letter. Guidelines for resubmitting your figure files are available below the reviewer comments at the end of this letter.

If applicable, we recommend that you deposit your laboratory protocols in protocols.io to enhance the reproducibility of your results. Protocols.io assigns your protocol its own identifier (DOI) so that it can be cited independently in the future. For instructions see: https://journals.plos.org/plosone/s/submission-guidelines#loc-laboratory-protocols. Additionally, PLOS ONE offers an option for publishing peer-reviewed Lab Protocol articles, which describe protocols hosted on protocols.io. Read more information on sharing protocols at https://plos.org/protocols?utm_medium=editorial-email&utm_source=authorletters&utm_campaign=protocols.

As the corresponding author, your ORCID iD is verified in the submission system and will appear in the published article. PLOS supports the use of ORCID, and we encourage all coauthors to register for an ORCID iD and use it as well. Please encourage your coauthors to verify their ORCID iD within the submission system before final acceptance, as unverified ORCID iDs will not appear in the published article. *Only*  the individual author can complete the verification step; PLOS staff *cannot*  verify ORCID iDs on behalf of authors.

We look forward to receiving your revised manuscript.

Kind regards,

Godwin Banafo Akrong, Ph.D.

Academic Editor

PLOS One

Journal Requirements:

If the reviewer comments include a recommendation to cite specific previously published works, please review and evaluate these publications to determine whether they are relevant and should be cited. There is no requirement to cite these works unless the editor has indicated otherwise.

Please review your reference list to ensure that it is complete and correct. If you have cited papers that have been retracted, please include the rationale for doing so in the manuscript text, or remove these references and replace them with relevant current references. Any changes to the reference list should be mentioned in the rebuttal letter that accompanies your revised manuscript. If you need to cite a retracted article, indicate the article’s retracted status in the References list and also include a citation and full reference for the retraction notice.

[Note: HTML markup is below. Please do not edit.]

Reviewers' comments:

Reviewer's Responses to Questions

**Comments to the Author**

Reviewer #1: All comments have been addressed

Reviewer #3: (No Response)

**********

2. Is the manuscript technically sound, and do the data support the conclusions?

Reviewer #1: Yes

Reviewer #3: Partly

**********

3. Has the statistical analysis been performed appropriately and rigorously?

Reviewer #1: Yes

Reviewer #3: (No Response)

**********

4. Have the authors made all data underlying the findings in their manuscript fully available?

The PLOS Data policy

Reviewer #1: Yes

Reviewer #3: No

**********

5. Is the manuscript presented in an intelligible fashion and written in standard English?

Reviewer #1: Yes

Reviewer #3: (No Response)

**********

Reviewer #1: The authors, Zhang et al., have adequately addressed all the comments and concerns raised during the initial review process. The manuscript has been revised accordingly, and the revisions have significantly improved its quality and clarity. I am satisfied with the authors’ responses and would strongly recommend the manuscript for consideration in the next stage of the publication process.

Reviewer #3: Journal: PLOS ONE

MS ID: PONE-D-25-68043R1

Title: Quantitative Assessment of China's Policies on the Online Sales of Prescription Drugs

Version: R1 Date: 22 June 2026

Reviewer's report:

Comment 1—The manuscript contains 'Error! Reference source not found.' placeholders in the text (at lines 49 and 61). The authors must update all cross-references and field codes, perform a global search for 'Error!' and broken citations, and verify that the final PDF renders correctly before resubmission.

Comment 2—Clarify threshold robustness further (Reviewer 2, Point 1).

Comment 3—Report the coherence scores for the 3-topics solution to demonstrate that the LDA model is not merely statistically convenient but semantically meaningful.

Comment 4—Section 4.4 (Indicator Weight Analysis) appears after Section 4.5 (LDA Model Analysis). The authors must reorder the manuscript to ensure that all Section 4.x subsections appear consecutively within the Results section.

Comment 5—A brief justification of why X2, X3, X4, and X10 variables are retained in the PMC index, despite having no informational variance for weighting, would strengthen the methodology.

Comment 6—The language of the text mining is ambiguous, and the authors have not explicitly stated whether the original Chinese policy documents or English-translated versions were analyzed.

Comment 7—A final, meticulous proofread is absolutely recommended, with particular attention to: (1) grammatical errors and awkward phrasing throughout; (2) inconsistent notation in equations (especially the index j in the Xtj term of Equation 2); and (3) broken cross-references to figures and tables.

Comment 8—According to PLOS ONE's policy, the "minimal data set" includes the data required to replicate all study findings. The supplementary materials provided are insufficient to fully support the authors' Data Availability Statement that all data are fully available without restriction.

**********

what does this mean?). If published, this will include your full peer review and any attached files.

If you choose “no”, your identity will remain anonymous but your review may still be made public.

**Do you want your identity to be public for this peer review?**  For information about this choice, including consent withdrawal, please see our Privacy Policy

Reviewer #1: **Yes:** Kingsley Amoafo

Reviewer #3: **Yes:** Mahmoud A. Abdel-Fattah

**********

[NOTE: If reviewer comments were submitted as an attachment file, they will be attached to this email and accessible via the submission site. Please log into your account, locate the manuscript record, and check for the action link "View Attachments". If this link does not appear, there are no attachment files.]

To ensure your figures meet our technical requirements, please review our figure guidelines: https://journals.plos.org/plosone/s/figures

You may also use PLOS’s free figure tool, NAAS, to help you prepare publication quality figures: https://journals.plos.org/plosone/s/figures#loc-tools-for-figure-preparation.

NAAS will assess whether your figures meet our technical requirements by comparing each figure against our figure specifications.

# Author comment: Author response to Decision Letter 2

**DOI:** 10.1371/journal.pone.0358484.r004

10 Jul 2026

Reviewer 3| 29 June 2026 | 16:34

#1. The manuscript contains 'Error! Reference source not found.' placeholders in the text (at lines 49 and 61). The authors must update all cross-references and field codes, perform a global search for 'Error!' and broken citations, and verify that the final PDF renders correctly before resubmission.

[Authors’ response] We sincerely apologize for the formatting oversights in our manuscript and greatly appreciate the reviewer’s valuable suggestions for revision. In response to the identified formatting issues, we have carefully revised all incorrect cross-references and domain codes throughout the manuscript. Additionally, we have regenerated the PDF file and thoroughly double-checked all citations to ensure complete accuracy and normal display in the revised version.

#2. Clarify threshold robustness further (Reviewer 2, Point 1).

[Authors’ response] We sincerely appreciate the reviewer’s suggestion to elaborate on threshold robustness. Following your advice, we conducted multiple rounds of sensitivity tests by integrating our policy sample data with widely accepted threshold criteria adopted in previous policy evaluation studies to validate the robustness of our threshold settings. We have further refined the relevant explanations regarding threshold robustness in the revised manuscript and updated the corresponding content.

<1>We have further supplemented and elaborated on the robustness of our threshold settings in Section 3.2.

Based on the characteristics of the policy samples and established policy evaluation criteria from prior studies[23,25], the stability and rationality of the threshold values were verified through multiple sensitivity tests to develop the PMC index grading standard for policy evaluation in this study. (See lines 171-173)

#3. Report the coherence scores for the 3-topics solution to demonstrate that the LDA model is not merely statistically convenient but semantically meaningful.

[Authors’ response] We sincerely appreciate the reviewer’s valuable and constructive comments. Following your suggestions, we have supplemented the topic coherence scores for the three-topic LDA model. These metrics confirm that the selected LDA model is not only statistically robust but also delivers practical semantic value in interpreting policy texts. In direct response to your specific requirements, we have made the following comprehensive changes:

<1>We have incorporated the coherence scores for the three topics into Table 5 in Section 4.5, which further verifies the rationality of our chosen number of topics.

The optimal number of topics was determined using two evaluation metrics: the perplexity curve and the topic coherence score. The model achieved an overall average topic coherence score of 0.46. This value falls within a reasonable range due to the limited sample size of policy texts in this study. Combined with the global minimum perplexity value and the semantic interpretability of feature keywords for each topic, three was ultimately selected as the optimal number of topics. (See lines 335-340)

Table 5.

Topic - Feature Words of the 18 Policy Texts

Theme Feature Words Weight /% Coherence Score

Qualification Supervision of Online Pharmaceutical Sales Internet, Pharmaceuticals, Websites, Operators, Consumers, Filing, Qualification Certificates, Laws 44.6

0.41

Control of Online Prescription Drug Sales Platforms, Fines, Medical Institutions, Inspections, Prescriptions, Violations, Sellers, Supervision 27.2

0.42

Construction of Smart Regulatory System Internet, Reform, Fields, Platforms, Construction, Data, Technology, Supervision, Optimization, Industry, Institutions, System

28.2

0.54

(See lines 358-360)

#4. Section 4.4 (Indicator Weight Analysis) appears after Section 4.5 (LDA Model Analysis). The authors must reorder the manuscript to ensure that all Section 4.x subsections appear consecutively within the Results section.

[Authors’ response] We sincerely apologize for the sequential formatting errors in our submitted manuscript and appreciate the reviewer’s thoughtful revision suggestions. To address the formatting issues you raised, we have reordered Sections 4.4 (Indicator Weight Analysis) and 4.5 (LDA Model Analysis) in the revised manuscript.

#5. A brief justification of why X2, X3, X4, and X10 variables are retained in the PMC index, despite having no informational variance for weighting, would strengthen the methodology.

[Authors’ response] We sincerely appreciate the reviewer’s insightful and constructive comments. We hereby elaborate on why variables X2, X3, X4, and X10 have been retained despite exhibiting insignificant variance during PMC index calculation. These four indicators respectively represent policy constraint intensity, issuing authority tier, legal validity level, and policy disclosure requirements, which serve as core dimensions for assessing the legality and authority of regulatory policies governing the online sales of prescription drugs. Excluding these variables would lead to the absence of key dimensions in the evaluation framework and hinder comprehensive quantitative policy assessment. To guarantee the objectivity and comparability of the horizontal comparative analysis of PMC indices across different policies in Section 4.2, we have retained these four variables and supplemented the corresponding justifications in the methodology section. In direct response to your specific requirements, we have made the following comprehensive changes:

<1>In Section 3.1, this study systematically elaborates on the necessity of including each evaluation indicator in the assessment system.

Aiming to ensure the integrity and rigor of the evaluation system and perform comprehensive quantitative assessments of the included policies, this study established a multidimensional quantitative policy evaluation framework comprising 10 primary variables and 35 secondary variables, as detailed in Table 2. (See lines 149-152)

#6. The language of the text mining is ambiguous, and the authors have not explicitly stated whether the original Chinese policy documents or English-translated versions were analyzed.

[Authors’ response]We appreciate the reviewer for raising this valuable comment. We acknowledge that the description of data sources used for policy text mining was insufficiently clear in the original manuscript. Herein, we clarify that the original Chinese versions of policy documents were selected as analytical materials for text mining in this study. All relevant keywords and professional terminology were translated with reference to authoritative English legal documents such as the Drug Administration Law of the People's Republic of China to ensure translation accuracy. We have supplemented detailed descriptions in the revised manuscript to clearly elaborate on the above information.

<1>We have added relevant clarifications in Section 2.2 (Analysis of Policy Text Mining) to specify that all materials used in this study are the original Chinese versions of policy documents.

In this study, ROST CM6.0 software was used to conduct in-depth text mining analysis of the original Chinese versions of policies governing the online sale of prescription drugs. (See lines 98-99)

#7. A final, meticulous proofread is absolutely recommended, with particular attention to: (1) grammatical errors and awkward phrasing throughout; (2) inconsistent notation in equations (especially the index j in the Xtj term of Equation 2); and (3) broken cross-references to figures and tables.

[Authors’ response] We sincerely apologize for the grammatical and formatting oversights in the original manuscript. We are grateful for your meticulous review and the careful identification of these issues. We have conducted a thorough final proofreading of the entire manuscript, revising grammatical errors, inconsistent formula symbols, and invalid cross-references for all figures and tables throughout the text.

#8. According to PLOS ONE's policy, the "minimal data set" includes the data required to replicate all study findings. The supplementary materials provided are insufficient to fully support the authors' Data Availability Statement that all data are fully available without restriction.

[Authors’ response] We sincerely appreciate the reviewer’s careful and valuable comments. In response to the issue that the currently submitted supplementary materials fail to support the statement of unrestricted public access to all data in our data availability section and do not meet the PLOS ONE data policy requirements for a minimal dataset that can fully reproduce all research findings, we have made the following revisions: First, we have reorganized the minimal dataset that can completely replicate all the research results in strict accordance with PLOS ONE’s data policy and supplemented these data into the supplementary materials, so that other researchers can repeat our entire empirical process and reproduce all study conclusions based on the submitted dataset. Second, we have revised the data availability statement accordingly. We removed the wording regarding unrestricted public access to all data and accurately described the scope of publicly available data and access permissions based on the newly uploaded minimal dataset, ensuring that the data availability statement is fully consistent with the supplementary materials we submitted.

Attachment Submitted filename: renamed_929a5.docx

# Peer review: Decision Letter 2

**DOI:** 10.1371/journal.pone.0358484.r005

**Authors:** Godwin Banafo Akrong

6 Aug 2026

Dear Dr.  Xie,

Thank you for submitting your manuscript to PLOS ONE. After careful consideration, we feel that it has merit but does not fully meet PLOS ONE’s publication criteria as it currently stands. Therefore, we invite you to submit a revised version of the manuscript that addresses the points raised during the review process.

Please submit your revised manuscript by Sep 20 2026 11:59PM. If you will need more time than this to complete your revisions, please reply to this message or contact the journal office at plosone@plos.org. When you're ready to submit your revision, log on to https://www.editorialmanager.com/pone/ and select the 'Submissions Needing Revision' folder to locate your manuscript file.

- A letter that responds to each point raised by the academic editor and reviewer(s). You should upload this letter as a separate file labeled 'Response to Reviewers'.
- A marked-up copy of your manuscript that highlights changes made to the original version. You should upload this as a separate file labeled 'Revised Manuscript with Track Changes'.
- An unmarked version of your revised paper without tracked changes. You should upload this as a separate file labeled 'Manuscript'.

If you would like to make changes to your financial disclosure, please include your updated statement in your cover letter. Guidelines for resubmitting your figure files are available below the reviewer comments at the end of this letter.

If applicable, we recommend that you deposit your laboratory protocols in protocols.io to enhance the reproducibility of your results. Protocols.io assigns your protocol its own identifier (DOI) so that it can be cited independently in the future. For instructions see: https://journals.plos.org/plosone/s/submission-guidelines#loc-laboratory-protocols. Additionally, PLOS ONE offers an option for publishing peer-reviewed Lab Protocol articles, which describe protocols hosted on protocols.io. Read more information on sharing protocols at https://plos.org/protocols?utm_medium=editorial-email&utm_source=authorletters&utm_campaign=protocols.

As the corresponding author, your ORCID iD is verified in the submission system and will appear in the published article. PLOS supports the use of ORCID, and we encourage all coauthors to register for an ORCID iD and use it as well. Please encourage your coauthors to verify their ORCID iD within the submission system before final acceptance, as unverified ORCID iDs will not appear in the published article. *Only*  the individual author can complete the verification step; PLOS staff *cannot*  verify ORCID iDs on behalf of authors.

We look forward to receiving your revised manuscript.

Kind regards,

Godwin Banafo Akrong, Ph.D.

Academic Editor

PLOS One

Journal Requirements:

1. If the reviewer comments include a recommendation to cite specific previously published works, please review and evaluate these publications to determine whether they are relevant and should be cited. There is no requirement to cite these works unless the editor has indicated otherwise.

2. Please review your reference list to ensure that it is complete and correct. If you have cited papers that have been retracted, please include the rationale for doing so in the manuscript text, or remove these references and replace them with relevant current references. Any changes to the reference list should be mentioned in the rebuttal letter that accompanies your revised manuscript. If you need to cite a retracted article, indicate the article’s retracted status in the References list and also include a citation and full reference for the retraction notice.

Additional Editor Comments:

I encourage you to address the comments raised by Reviewer #1 and make the necessary revisions. I look forward to reviewing your revised manuscript.

[Note: HTML markup is below. Please do not edit.]

Reviewer's Responses to Questions

**Comments to the Author**

Reviewer #1: All comments have been addressed

Reviewer #3: All comments have been addressed

**********

2. Is the manuscript technically sound, and do the data support the conclusions?

Reviewer #1: No

Reviewer #3: Yes

**********

3. Has the statistical analysis been performed appropriately and rigorously?

Reviewer #1: No

Reviewer #3: Yes

**********

4. Have the authors made all data underlying the findings in their manuscript fully available?

The PLOS Data policy

Reviewer #1: Yes

Reviewer #3: Yes

**********

5. Is the manuscript presented in an intelligible fashion and written in standard English?

Reviewer #1: Yes

Reviewer #3: Yes

**********

Reviewer #1: Recommendation 1: Threshold robustness

The added sentence in Section 3.2 only asserts that thresholds were validated "through multiple sensitivity tests" without showing any of the actual results. The authors should add concrete, verifiable content to Section 3.2, such as:

1. The specific threshold values tested (e.g., alternative cut-points for "Excellent," "Good," etc.) and the source studies each was drawn from.

2. The outcome of the sensitivity tests, for example, a table or brief narrative showing how many policies would shift classification (and by how much) under each alternative threshold set, demonstrating that the final grading bands are stable.

3. A brief explanation of the criterion used to select the final thresholds over the alternatives (e.g., minimal reclassification, best alignment with sample distribution, closest match to prior literature).

Recommendation 2: Justification for retaining X2, X3, X4, X10

The rationale given in the response letter (that these four variables represent policy constraint intensity, issuing authority tier, legal validity level, and policy disclosure requirements, and are core to assessing legality/authority) needs to actually be inserted into Section 3.1 of the manuscript it currently only appears in the response letter.

Reviewer #3: Journal: PLOS ONE

MS ID: PONE-D-25-68043R2

Title: Quantitative Assessment of China's Policies on the Online Sale of Prescription Drugs

Version: R2 Date: 26 July 2026

Reviewer's report:

After careful review of the revised manuscript (R2) and the authors' comprehensive responses to my previous comments, I am pleased to recommend acceptance of this manuscript for publication in its current form.

**********

what does this mean?). If published, this will include your full peer review and any attached files.

If you choose “no”, your identity will remain anonymous but your review may still be made public.

**Do you want your identity to be public for this peer review?**  For information about this choice, including consent withdrawal, please see our Privacy Policy

Reviewer #1: **Yes:** Kingsley Amoafo

Reviewer #3: **Yes:** Mahmoud A. Abdel-Fattah

**********

[NOTE: If reviewer comments were submitted as an attachment file, they will be attached to this email and accessible via the submission site. Please log into your account, locate the manuscript record, and check for the action link "View Attachments". If this link does not appear, there are no attachment files.]

To ensure your figures meet our technical requirements, please review our figure guidelines: https://journals.plos.org/plosone/s/figures

You may also use PLOS’s free figure tool, NAAS, to help you prepare publication quality figures: https://journals.plos.org/plosone/s/figures#loc-tools-for-figure-preparation.

NAAS will assess whether your figures meet our technical requirements by comparing each figure against our figure specifications.

Attachment Submitted filename: comments.docx

# Author comment: Author response to Decision Letter 3

**DOI:** 10.1371/journal.pone.0358484.r006

25 Aug 2026

Reviewer 1

#1: Recommendation 1: Threshold robustness

The added sentence in Section 3.2 only asserts that thresholds were validated "through multiple sensitivity tests" without showing any of the actual results. The authors should add concrete, verifiable content to Section 3.2, such as:

1. The specific threshold values tested (e.g., alternative cut-points for "Excellent," "Good," etc.) and the source studies each was drawn from.

2. The outcome of the sensitivity tests, for example, a table or brief narrative showing how many policies would shift classification (and by how much) under each alternative threshold set, demonstrating that the final grading bands are stable.

3. A brief explanation of the criterion used to select the final thresholds over the alternatives (e.g., minimal reclassification, best alignment with sample distribution, closest match to prior literature).

[Authors’ response] We sincerely appreciate the reviewer’s insightful and constructive comments. This study developed the grading standard for the PMC index based on the two core cluster centers, which were derived from cluster analysis of PMC index results using SPSS 27.0, and evaluation criteria reported in prior literature. In addition, we have systematically supplemented the sensitivity test results in Section 3.2 to fully verify the rationality and robustness of this grading standard. The detailed revisions are presented below.

<1>Add detailed empirical results of sensitivity tests in Section 3.2 to verify the robustness of the grading standard.

Based on the two core cluster centers (5.81, 6.75), which were derived from cluster analysis of PMC index results using SPSS 27.0, and policy evaluation criteria proposed in prior literature[23,25], this study developed a grading standard for the PMC index: PMC index values from 8.50 to 10.00 correspond to "Perfect", 6.50–8.49 to "Excellent", 5.00–6.49 to "Good", and 0.00–4.99 to "Non-compliant". To test the robustness of this grading standard, two alternative schemes with moderately adjusted cut-points around the baseline critical values were constructed for sensitivity analysis. Scheme A: Perfect [8.30, 10.00], Excellent [6.30, 8.29], Good [4.80, 6.29], Non-compliant [0.00, 4.79]; Scheme B: Perfect [8.70, 10.00], Excellent [6.70, 8.69], Good [5.20, 6.69], Non-compliant [0.00, 5.19]. The results indicate that three policies experience grade shifts under Scheme A, while only one policy undergoes a grade adjustment under Scheme B. No samples fall into the Perfect or Non-compliant intervals. The low proportion of sample reclassification across alternative schemes demonstrates that the defined grading bands exhibit satisfactory robustness. Therefore, the original threshold set is adopted as the grading criterion for policy evaluation in this study. (See lines 174-186)

Recommendation 2: Justification for retaining X2, X3, X4, X10

The rationale given in the response letter (that these four variables represent policy constraint intensity, issuing authority tier, legal validity level, and policy disclosure requirements, and are core to assessing legality/authority) needs to actually be inserted into Section 3.1 of the manuscript it currently only appears in the response letter.

[Authors’ response] We greatly appreciate the reviewer’s valuable suggestion. We fully agree with this comment and have formally incorporated the rationale for retaining variables X2, X3, X4, and X10 into the revised manuscript.

<1>In Section 3.1, this study systematically elaborates on the necessity of including each evaluation indicator in the assessment system.

Among these indicators, X2, X3, X4, and X10 respectively represent policy constraint intensity, issuing authority tier, legal validity level, and policy disclosure requirements, which serve as core dimensions for assessing the legality and authority of regulatory policies governing the online sales of prescription drugs. (See lines 149-152)

Attachment Submitted filename: Response letter to Reviewer 1.docx

# Peer review: Decision Letter 3

**DOI:** 10.1371/journal.pone.0358484.r007

**Authors:** Godwin Banafo Akrong

1 Sep 2026

Quantitative Assessment of China's Policies on the Online Sale of Prescription Drugs

PONE-D-25-68043R3

Dear Dr. Xue-Feng,

We’re pleased to inform you that your manuscript has been judged scientifically suitable for publication and will be formally accepted for publication once it meets all outstanding technical requirements.

Within one week, you’ll receive an e-mail detailing the required amendments. When these have been addressed, you’ll receive a formal acceptance letter and your manuscript will be scheduled for publication.

An invoice will be generated when your article is formally accepted. Please note, if your institution has a publishing partnership with PLOS and your article meets the relevant criteria, all or part of your publication costs will be covered. Please make sure your user information is up-to-date by logging into Editorial Manager at Editorial Manager® and clicking the ‘Update My Information' link at the top of the page. For questions related to billing, please contact billing support.

If your institution or institutions have a press office, please notify them about your upcoming paper to help maximize its impact. If they’ll be preparing press materials, please inform our press team as soon as possible -- no later than 48 hours after receiving the formal acceptance. Your manuscript will remain under strict press embargo until 2 pm Eastern Time on the date of publication. For more information, please contact onepress@plos.org.

Kind regards,

Godwin Banafo Akrong, Ph.D.

Academic Editor

PLOS One

Additional Editor Comments (optional):

Reviewers' comments:

Reviewer's Responses to Questions

**Comments to the Author**

Reviewer #1: All comments have been addressed

Reviewer #3: All comments have been addressed

**********

2. Is the manuscript technically sound, and do the data support the conclusions?

Reviewer #1: Yes

Reviewer #3: Yes

**********

3. Has the statistical analysis been performed appropriately and rigorously?

Reviewer #1: Yes

Reviewer #3: Yes

**********

4. Have the authors made all data underlying the findings in their manuscript fully available?

The PLOS Data policy

Reviewer #1: Yes

Reviewer #3: Yes

**********

5. Is the manuscript presented in an intelligible fashion and written in standard English?

Reviewer #1: Yes

Reviewer #3: Yes

**********

Reviewer #1: (No Response)

Reviewer #3: Journal: PLOS ONE

MS ID: PONE-D-25-68043R3

Title: Quantitative Assessment of China's Policies on the Online Sale of Prescription Drugs

Version: R3 Date: 28 August 2026

Reviewer's report:

All my previous comments have been addressed.

**********

what does this mean?). If published, this will include your full peer review and any attached files.

If you choose “no”, your identity will remain anonymous but your review may still be made public.

**Do you want your identity to be public for this peer review?**  For information about this choice, including consent withdrawal, please see our Privacy Policy

Reviewer #1: **Yes:** Kingsley Amoafo

Reviewer #3: **Yes:** Mahmoud A. Abdel-Fattah

**********

# Editor report: Acceptance letter

**DOI:** 10.1371/journal.pone.0358484.r008

**Authors:** Godwin Banafo Akrong

PONE-D-25-68043R3

PLOS One

Dear Dr. Xie,

I'm pleased to inform you that your manuscript has been deemed suitable for publication in PLOS One. Congratulations! Your manuscript is now being handed over to our production team.

At this stage, our production department will prepare your paper for publication. This includes ensuring the following:

* All references, tables, and figures are properly cited

* All relevant supporting information is included in the manuscript submission,

* There are no issues that prevent the paper from being properly typeset

You will receive further instructions from the production team, including instructions on how to review your proof when it is ready. Please keep in mind that we are working through a large volume of accepted articles, so please give us a few days to review your paper and let you know the next and final steps.

Lastly, if your institution or institutions have a press office, please let them know about your upcoming paper now to help maximize its impact. If they'll be preparing press materials, please inform our press team within the next 48 hours. Your manuscript will remain under strict press embargo until 2 pm Eastern Time on the date of publication. For more information, please contact onepress@plos.org.

You will receive an invoice from PLOS for your publication fee after your manuscript has reached the completed accept phase. If you receive an email requesting payment before acceptance or for any other service, this may be a phishing scheme. Learn how to identify phishing emails and protect your accounts at https://explore.plos.org/phishing.

If we can help with anything else, please email us at customercare@plos.org.

Thank you for submitting your work to PLOS One and supporting open access.

Kind regards,

PLOS One Editorial Office Staff

on behalf of

Dr. Godwin Banafo Akrong

Academic Editor

PLOS One
